# CHIP mutations affect the heat shock response differently in human fibroblasts and iPSC-derived neurons

**DOI:** 10.1242/dmm.045096

**Published:** 2020-10-12

**Authors:** S. Schuster, E. Heuten, A. Velic, J. Admard, M. Synofzik, S. Ossowski, B. Macek, S. Hauser, L. Schöls

**Affiliations:** 1 Department of Neurology and Hertie Institute for Clinical Brain Research, University of Tübingen, 72076 Tübingen, Germany; 2 Department of Neurodegenerative Diseases, German Center for Neurodegenerative Diseases (DZNE), 72076 Tübingen, Germany; 3 Graduate School of Cellular and Molecular Neuroscience, University of Tübingen, 72076 Tübingen, Germany; 4 Proteome Center Tübingen, University of Tübingen, 72076 Tübingen, Germany; 5 Institute for Medical Genetics and Applied Genomics, University of Tübingen, 72076 Tübingen, Germany

**Keywords:** CHIP/STUB1, Cortical neurons, CRISPR/Cas9, Heat shock response, Induced pluripotent stem cells, SCAR16

## Abstract

C-terminus of HSC70-interacting protein (CHIP) encoded by the gene *STUB1* is a co-chaperone and E3 ligase that acts as a key regulator of cellular protein homeostasis. Mutations in *STUB1* cause autosomal recessive spinocerebellar ataxia type 16 (SCAR16) with widespread neurodegeneration manifesting as spastic-ataxic gait disorder, dementia and epilepsy. CHIP^−/−^ mice display severe cerebellar atrophy, show high perinatal lethality and impaired heat stress tolerance. To decipher the pathomechanism underlying SCAR16, we investigated the heat shock response (HSR) in primary fibroblasts of three SCAR16 patients. We found impaired HSR induction and recovery compared to healthy controls. *HSPA1A/B* transcript levels (coding for HSP70) were reduced upon heat shock but HSP70 remained higher upon recovery in patient- compared to control-fibroblasts. As SCAR16 primarily affects the central nervous system we next investigated the HSR in cortical neurons (CNs) derived from induced pluripotent stem cells of SCAR16 patients. We found CNs of patients and controls to be surprisingly resistant to heat stress with high basal levels of HSP70 compared to fibroblasts. Although heat stress resulted in strong transcript level increases of many HSPs, this did not translate into higher HSP70 protein levels upon heat shock, independent of *STUB1* mutations. Furthermore, *STUB1*(−/−) neurons generated by CRISPR/Cas9-mediated genome editing from an isogenic healthy control line showed a similar HSR to patients. Proteomic analysis of CNs showed dysfunctional protein (re)folding and higher basal oxidative stress levels in patients. Our results question the role of impaired HSR in SCAR16 neuropathology and highlight the need for careful selection of proper cell types for modeling human diseases.

## Abstract (precis)

**Summary:** Cell viability, expression and immunocytochemical analyses reveal that mutations in *STUB1* that cause SCAR16 impair the heat-shock response in patient-derived fibroblasts, but not in iPSC-derived cortical neurons.

## INTRODUCTION

Mutations in *STUB1* (STIP1 homology and U-box containing protein 1) cause early-onset autosomal recessive cerebellar ataxia type 16 (OMIM 615768), a movement disorder that is characterized by atrophy of the cerebellum, brainstem and spinal cord, leading to loss of muscle coordination, unsteady gait, impaired fine motor skills and slurred speech (Shi et al., 2013; Bettencourt et al., 2015). Recently, patients with *STUB1* ataxia were shown to present a broader neurodegeneration with complex clinical phenotypes of cognitive impairment, epilepsy and hypogonadism in addition to spastic-ataxic movement disorder (Shi et al., 2013; Heimdal et al., 2014; Shi et al., 2014; Synofzik et al., 2014; Hayer et al., 2017).

*STUB1* codes for the C-terminus of HSC70-interacting protein (CHIP), which plays an important role in protein quality control. Its two major functions are linked to two distinct structural domains: via its tetratricopeptide repeat (TPR) domain, CHIP acts as a co-chaperone of HSC70/HSP70 and HSP90 and inhibits ATPase and refolding activity (Ballinger et al., 1999); and via its U box domain, it acts as an E3 ligase tagging chaperone-bound substrates, as well as tagging other substrates with ubiquitin (Jiang et al., 2001; Murata et al., 2003). Degradation of those proteins or organelles occurs via the ubiquitin-proteasome system (UPS) or the autophagy-lysosome system (Zhang et al., 2005; Yao, 2010; Guo et al., 2015). Both mechanisms play a major role in protein quality control and sustain proper cellular homeostasis.

More recently, CHIP was identified as a regulator of many other processes, such as TFEB activity and thereby macroautophagy regulation (Guo et al., 2015; Sha et al., 2017), necroptosis (Seo et al., 2016; Tang et al., 2018), cAMP and AMPK signaling (Schisler et al., 2013; Rinaldi et al., 2019), oxidative metabolism (Ravi et al., 2018), chaperone-mediated autophagy (Ferreira et al., 2015) and neuronal preconditioning (Lizama et al., 2018). In addition to this, CHIP was shown to be cytoprotective in many forms of neurodegeneration by degrading *inter alia* α-synuclein (Shin et al., 2005; Tetzlaff et al., 2008), LRRK2 (Ding and Goldberg, 2009), APP and BACE1 (Kumar et al., 2007; Singh and Pati, 2015), as well as huntingtin (Jana et al., 2005; Miller et al., 2005). This leads to the assumption that CHIP might be a key player in neurodegeneration and a promising target for the treatment of many neurodegenerative diseases. Furthermore, CHIP was previously linked to cardiac and muscular disorders and several different types of cancers (reviewed by Joshi et al., 2016).

CHIP expression is highest in tissues with high metabolic activity, i.e. heart, skeletal muscle and brain (Ballinger et al., 1999)*.* In mouse brains, CHIP is expressed primarily in neurons of the cerebellum, pons, medulla oblongata, hippocampus and cerebral cortex (Sahara et al., 2005; Anderson et al., 2010). CHIP^−/−^ mice display severe cerebellar atrophy specifically in the Purkinje cell layer, with a distinct motor impairment phenotype. Furthermore, CHIP knockout mice were shown to have decreased stress tolerance and increased age-related phenotypes leading to decreased lifespan and a gonadal dysfunction (Dai et al., 2003; Min et al., 2008; Shi et al., 2014). Upon thermal challenge, 100% of CHIP^−/−^ mice die during heat shock or shortly after (Dai et al., 2003), suggesting a key role for CHIP in stress response, and more specifically in heat shock response (HSR).

Environmental stressors such as heat, heavy metals and reactive oxygen species, but also pathophysiological stressors, such as protein aggregation, inflammation and tissue injury, can induce HSR, which is characterized by a rapid increase in the expression of heat shock proteins (reviewed by Richter et al., 2010). This induction of heat shock proteins is regulated by the key transcription factor heat shock factor 1 (HSF1). Under basal conditions, HSF1 is present in the cytoplasm in its inactive monomeric form, shielded by HSC70/HSP70 and HSP90 (Abravaya et al., 1991). Upon stress, the chaperones blocking HSF1 are recruited to the site of accumulating misfolded proteins, thereby releasing HSF1 (Santoro, 2000). HSF1 quickly trimerizes, undergoes post-translational modifications that modulate its activity and translocates to the nucleus (Sarge et al., 1993). Here, it binds to heat shock responsive elements, leading to the transcription of heat shock proteins, such as *HSPA1A/B* (encoding HSP70) and *DNAJB1* (encoding HSP40) that act to refold or clear misfolded proteins and confer cytoprotection (reviewed by Richter et al., 2010). Other small HSPs (sHSPs), such as HSPB1, HSPB5 and HSPB8 assist by binding to unfolded proteins to prevent aggregation (Acunzo et al., 2012). Once cellular homeostasis is re-established, free HSPs inhibit HSF1 and attenuate the HSR (Abravaya et al., 1991; Baler et al., 1992; Vjestica et al., 2013), allowing a tight regulation of protein quality control during stress conditions.

CHIP was shown to directly interact with HSF1 upon stress induction, co-translocating from the cytoplasm to the nucleus and thereby increasing HSP70 expression in non-neuronal cell types, such as fibroblasts, human embryonic kidney (HEK) cells and retinal epithelium cells, with no or a reduced response in CHIP^−/−^ or CHIP^mut^ cells (Dai et al., 2003; Kim et al., 2005; Qian et al., 2006). Furthermore, CHIP expression is required for HSP70 turnover upon recovery (Qian et al., 2006). Dai et al. (2003) also showed that HSP70 expression upon heat shock in CHIP^−/−^ mice was strongly reduced compared to wild type in most tissues, with an almost complete absence of HSP70 in brain, heart and spleen.

To understand the pathomechanism induced by mutant *STUB1*, we assessed the HSR in fibroblasts of three SCAR16 patients and three healthy controls and found an impaired HSR in patients. To reflect the central nervous system being the major focus of disease in SCAR16, we next investigated HSR in cortical neurons (CNs) generated from induced pluripotent stem cells (iPSCs). Although heat stress resulted in increased transcript levels of HSPs, this surprisingly did not translate into higher protein levels of HSP70. Proteomic analysis of patient and control CNs showed disturbances in protein folding, the ubiquitin system and oxidative stress response. Both approaches question the role of impaired HSR in SCAR16 neuropathology and highlight the need for the careful selection of proper cell types for disease modeling.

## RESULTS

### CHIP mutations do not alter viability of fibroblasts during prolonged heat stress

**Fig. 1.**
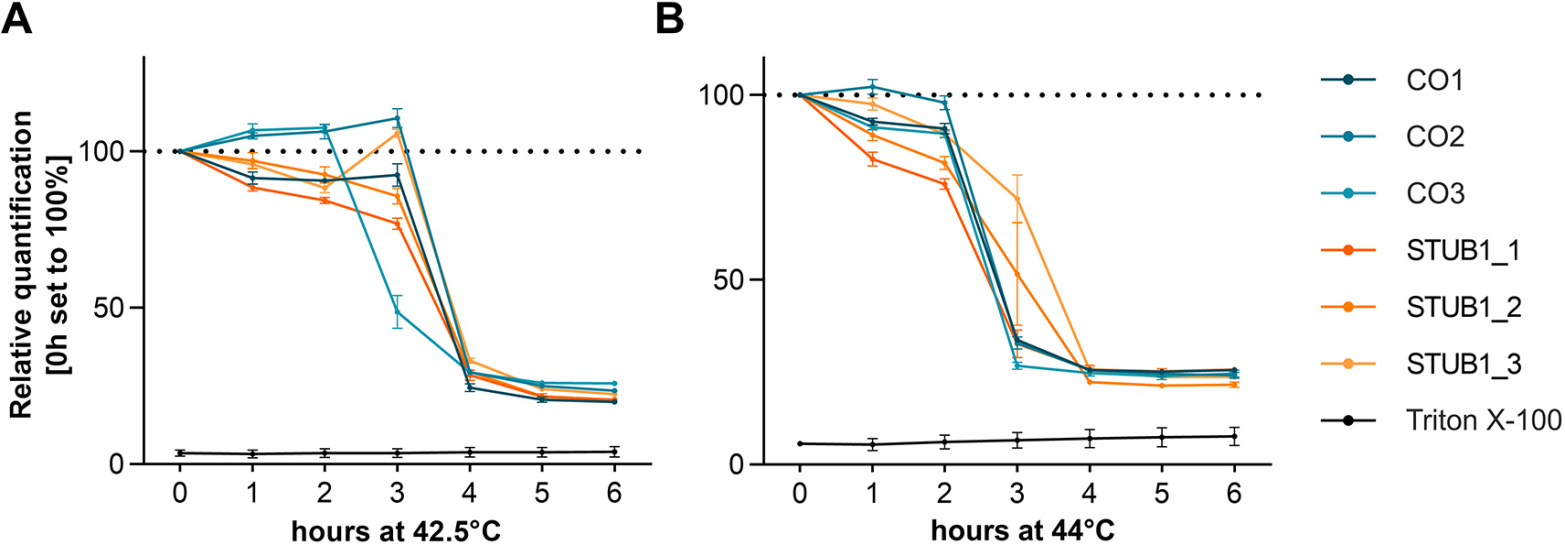
**Cell viability of fibroblasts upon heat stress is not impaired by dysfunctional CHIP.** (A,B) Viability of fibroblasts (%) during prolonged heat stress at 42.5°C (A) and 44°C (B) for 6 h of three controls and three *STUB1* patients. Data are mean±s.d. of triplicates. Triton X-100 was used as negative control for cell viability.

As CHIP has been linked to cell viability upon heat shock, we assessed the effect of prolonged heat stress on the viability of fibroblasts in three patients with *STUB1* mutations (STUB1_1, STUB1_2 and STUB1_3) in comparison to three healthy controls (CO1, CO2, CO3) (for further details see Table S1). CHIP protein levels were confirmed to be reduced in all three patient-derived fibroblasts compared to the three healthy controls (Fig. S1A). For cell viability analysis, we exposed cells to either 42.5°C or 44°C for 6 h and quantified the signal with the CyQuant direct cell proliferation assay. Cell viability decreased rapidly between 3 and 4 h of prolonged heat stress at 42.5°C [mean±s.e.m. (%); 3 h heat shock, 86.67±9.16; and 4 h heat shock, 29.01±1.15] (Fig. 1A). At 44°C, cell death mostly occurred after 2 to 3 h of maintained heat shock (2 h heat shock, 87.53±3.16; 3 h heat shock, 41.5±6.95; and 4 h heat shock, 24.88±0.54) (Fig. 1B). No consistent difference was detected between patients and controls. We therefore could not determine an effect of *STUB1* mutations on fibroblast viability upon prolonged heat stress.

### *STUB1* mutations cause impaired heat shock response induction and recovery in fibroblasts

Previous reports have shown that CHIP plays a role in both the induction and recovery of the HSR in HEK and HeLa cells, as well as fibroblasts (Dai et al., 2003; Kim et al., 2005; Qian et al., 2006; Zhang et al., 2015). To test whether CHIP mutations lead to dysfunctional HSR, we analyzed HSF1 translocation upon heat shock, HSR induction by transcript analysis and protein expression analysis and HSR recovery by protein analysis in three STUB1 patient- and three healthy age- and gender-matched control-derived fibroblasts.

**Fig. 2.**
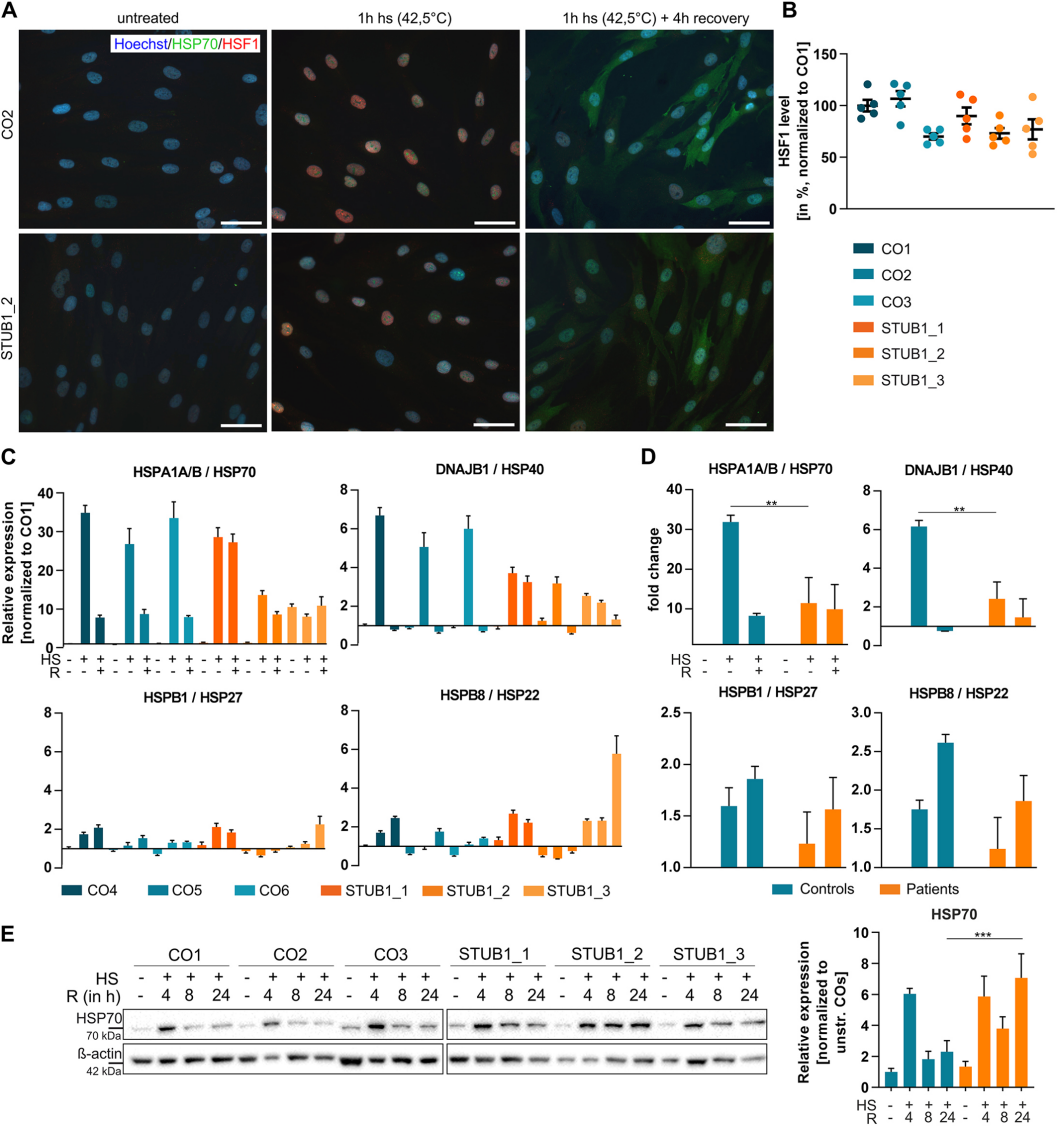
**Mutant CHIP impairs HSR induction and recovery in fibroblasts.** (A) Immunocytochemical staining of HSF1 (red), HSP70 (green) and with Hoechst (blue) in fibroblasts without stress, after heat shock (1 h, 42.5°C) and after recovery (4 h, 37°C). Exemplary images of one control and one patient are shown. Scale bars: 50 µm. (B) Quantification of HSF1 levels in nuclei of heat-shocked fibroblasts, given in percent and normalized to control line CO1. (C) Transcript analysis by qRT-PCR of *HSPA1A/B*, *DNAJB1*, *HSPB1* and *HSPB8* in unstressed, heat shocked (HS) and recovered (R) samples. Values are normalized to CO1 and the housekeeping genes *GAPDH* and *TBP*. *n*=3 replicates. (D) Pooled analysis of three controls and three patients. Transcript levels were normalized to the respective basal levels. (E) Western blot analysis of HSP70 protein levels in fibroblasts without stress, after 1 h heat shock and up to 24 h of recovery, with ß-actin as the loading control. One representative image out of three experiments is shown. Quantification is based on the normalization to unstressed controls. Three controls and three patients were pooled. Data are mean±s.e.m. **P<0.01, ***P<0.001, one-way ANOVA.

For the analysis of HSF1 translocation from cytoplasm to nucleus, we fixed cells after 1 h heat shock at 42.5°C and immunocytochemically stained for HSF1, HSP70 and with Hoechst 33258 (Fig. 2A). In all six cell lines (three patients, three controls), HSF1 and HSP70 proteins were barely detectable in unstressed conditions. Upon heat shock, HSF1 levels in the nuclei strongly increased and HSP70 accumulated in nuclear bodies. When 4 h of recovery at 37°C was added, HSF1 levels in the nuclei returned to baseline but cytosolic expression of HSP70 increased strongly. Representative images of two cell lines are shown in Fig. 2A. Upon quantification of nuclear HSF1 levels at 1 h of heat shock, we saw a trend towards lower HSF1 levels in patients compared to controls [mean±s.e.m. (%); controls, 92.3±5.0; and patients, 80.0±4.5; *P*=0.09] (Fig. 2B).

When transcripts of heat shock-related genes were analyzed, a strong increase of *HSPA1A/B (*coding for HSP70) and *DNAJB1* (coding for HSP40) was observed after 1 h of heat shock (Fig. 2C,D). However, this induction was significantly lower in patients compared to controls [mean±s.e.m.; *HSPA1A/B* in controls, 31.89±1.39-fold; and in patients, 11.44±5.25-fold (*P*=0.004). *DNAJB1* in controls, 6.17±0.25-fold; and in patients, 2.42 fold±0.71 (*P*=0.001)]. Levels in controls and patients decreased with an additional 4 h of recovery. *HSPB1* (coding for HSP27) and *HSPB8* (coding for HSP22) transcription slightly increased upon recovery [*HSPB1* in controls, 1.86±0.1-fold; and in patients, 1.56±0.25-fold. *HSPB8* in controls, 2.61±0.09-fold; and in patients, 1.86±0.27-fold (*P*=0.10)] (Fig. 2C,D). *HSP90AA1* (HSP90), *HSPA8* (HSC70) and *HSPA5* (BiP/GRP78) were neither strongly altered upon heat shock or recovery nor different between patients and controls (Fig. S1B,C).

In terms of protein levels, we saw very low levels of HSP70 under basal conditions in all cell lines and a strong induction of HSP70 at 1 h heat shock plus 4 h recovery (mean±s.e.m.; unstressed controls, 1±0.23; 1 h heat shock plus 4 h recovery; controls, 6.03±0.36; unstressed patients, 1.67±0.33; 1 h heat shock plus 4 h recovery; patients, 7.34±1.1) (Fig. 2E). Induction was 6.03-fold in controls and 4.39-fold in patients (*P*=0.1). However, HSP70 turnover, and thus HSR recovery, differed in controls and patients with prolonged recovery times, with significantly higher HSP70 levels after 24 h recovery in patients (controls, 2.88±0.76; patients, 7.07±1.55; *P*=0.0004).

In summary, we were able to show that HSF1 translocates to the nucleus upon heat shock in fibroblasts, with a slight but not significant reduction caused by *STUB1* mutations. This led to a significantly lower induction of *HSPA1A/B* and *DNAJB1* in patients. HSP70 protein levels were strongly induced at 1 h heat shock plus 4 h recovery compared to unstressed levels in both patients and controls, but levels in patients remained high at 8 h and 24 h after heat shock, indicating an impaired HSR recovery and HSP70 turnover.

### iPSC-derived CNs show typical morphology and express neuronal markers

**Fig. 3.**
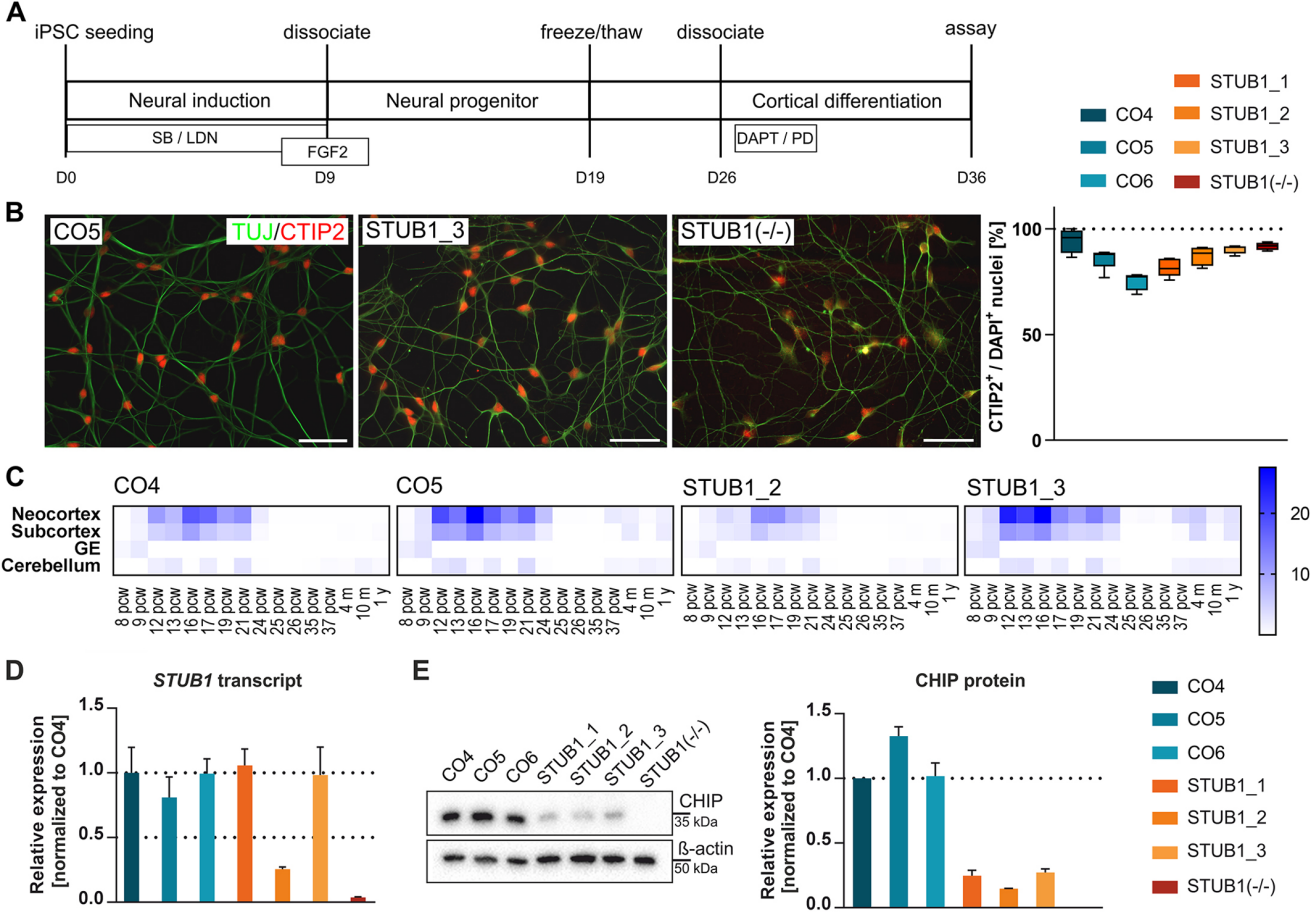
**Characterization of iPSC-derived neurons.** (A) Schematic representation of the experimental procedure of the differentiation of iPSCs into iPSC-derived CNs. SB, SB431542; LDN, LDN193189; PD, PD0325901. (B) Immunocytochemical stainings of CNs on D36 for TUJ (green) and CTIP2 (red). Scale bars: 50 µm. Exemplary stainings of three lines are shown. Quantification of the percentage of CTIP2^+^/DAPI^+^ cells in controls, patients and STUB1(−/−) was performed for four to five fields per cell line. Values are given as boxplots showing minimum to maximum, with the mean indicated by a line. (C) Transcript expression in CNs match the best expression found in neocortical tissue at postconception week 16. Heatmaps were produced for two controls (CO4 and CO5) and two patients (STUB1_2 and STUB1_3) by Wilcoxon rank-sum comparisons of CN transcripts to the BrainSpan atlas, and results are shown as –log 10 *P*-values of significant differences. All four generated lines display a similar expression pattern. (D) Transcript level of *STUB1* in three controls, three patients and the homozygous knockout line. Levels are normalized to CO4. Dotted lines indicate full transcript level and 50% reduced transcript level. (E) CHIP protein expression level was analyzed by western blotting. One representative blot is shown. Bands are quantified densitometrically and normalized to ß-actin and CO4. Data are mean±s.e.m. *n*=3. GE, ganglionic eminence; iPSC, induced pluripotent stem cells; pcw, postconception week.

As SCAR16 primarily affects the central nervous system, we next investigated the HSR in CNs. For this, we generated iPSCs from three SCAR16 patients (STUB1_1, STUB1_2 and STUB1_3) and three gender-matched healthy controls (CO4, CO5 and CO6) (for further details see Table S1). Genomic integrity and pluripotency were confirmed for all cell lines [data not shown; for details see Schuster et al. (2018)]. Additionally, we generated a *STUB1* knockout line STUB1(−/−) by CRISPR/Cas9-mediated genome editing of the isogenic control line CO5 (Schuster et al., 2019). iPSCs were differentiated into CNs according to a previously published protocol, with slight modifications (Shi et al., 2012; Rehbach et al., 2019) (Fig. 3A). After 36 days of differentiation, the cultures were highly homogeneous, with all cells being positive for ß-III-tubulin (TUJ) and with more than 75% of cells being positive for CTIP2 (also known as BCL11B) (mean±s.e.m., 86.7±1.3%), a cortical layer V marker (Fig. 3B). There was no significant difference in the percentage of CTIP2^+^ nuclei between *STUB1* patients, knockout and controls.

To assess the spatial and temporal identity of the generated CNs and to exclude differences in differentiation potential and state, we compared transcript analysis data of biological triplicates of CO4, CO5, STUB1_2 and STUB1_3 with transcript data from the developing human brain [BrainSpan atlas (www.brainspan.org) data from fetal and early childhood postmortem tissue up to 1 year of age]. The gene signature of both patient and control iPSC-derived CNs most significantly matched the co-expressed gene sets in the neocortex and the subcortex at postconception week (pcw) 12 to 21, with the strongest correlation at pcw 16 (Fig. 3C). The four analyzed cell lines display only slight differences in the expression patterns independent of *STUB1* mutations and show the same temporospatial pattern.

In generated CNs, we quantified *STUB1* transcript levels and CHIP protein expression levels. In terms of transcript levels, patients STUB1_1 and STUB1_3 showed no difference to the controls (Fig. 3D), whereas at the protein level, CHIP expression was reduced in the CNs of both patients to 25% of control levels (Fig. 3E). STUB1_2 neurons showed a reduced *STUB1* transcript level to 25% of controls (Fig. 3D), resulting in an even lower protein level of 15% compared to wild type (Fig. 3E). STUB1(−/−) showed a transcript level of 3% compared to controls (Fig. 3D), and no detectable protein (Fig. 3E), confirming the homozygous knockout of *STUB1.*

### CHIP mutations do not alter cell viability during prolonged heat stress in CNs

**Fig. 4.**
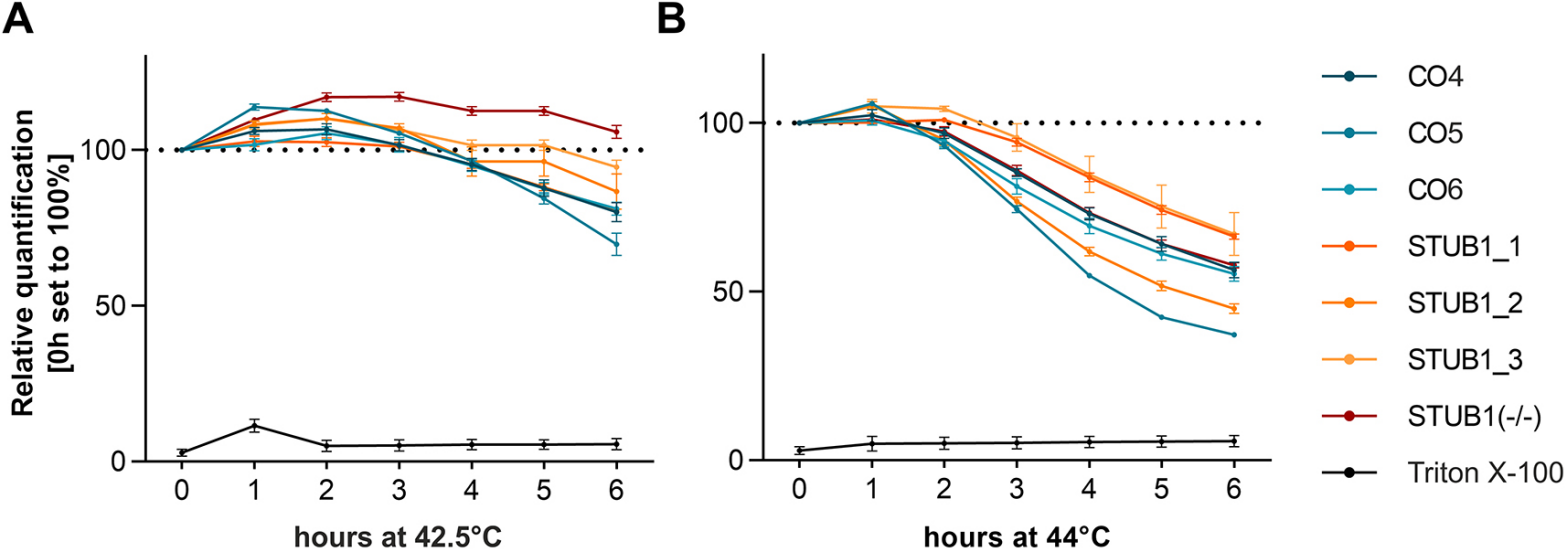
**Viability of CNs upon heat shock is not impaired by dysfunctional CHIP.** (A,B) Cell viability (%) during prolonged heat shock at 42.5°C (A) and 44°C (B) was investigated in three controls, three *STUB1* patients and a STUB1(−/−) line. Data are mean±s.d. of triplicates. Triton X-100 was used as negative control for cell viability.

To examine the effect of heat shock on the viability of CNs, we exposed CNs of three controls, three *STUB1* patients and STUB1(−/−) to prolonged heat stress at either 42.5°C or 44°C for 6 h. Interestingly, signal intensity of the CyQuant direct cell proliferation assay slightly increased in all cell lines at 42.5°C and only reached levels below baseline after more than 4 h of heat stress. After 6 h of heat stress, a mean value of 85.5±11.1% of viable cells was reached (Fig. 4A). At 44°C, lethality of CNs was higher, reaching a mean value of 55.0±10.3% of viable cells after 6 h of heat stress (Fig. 4B). No consistent difference in cell viability was observable between controls, patients and the STUB1(−/−) line.

### Mutations in *STUB1* do not impair the heat shock response in CNs

**Fig. 5.**
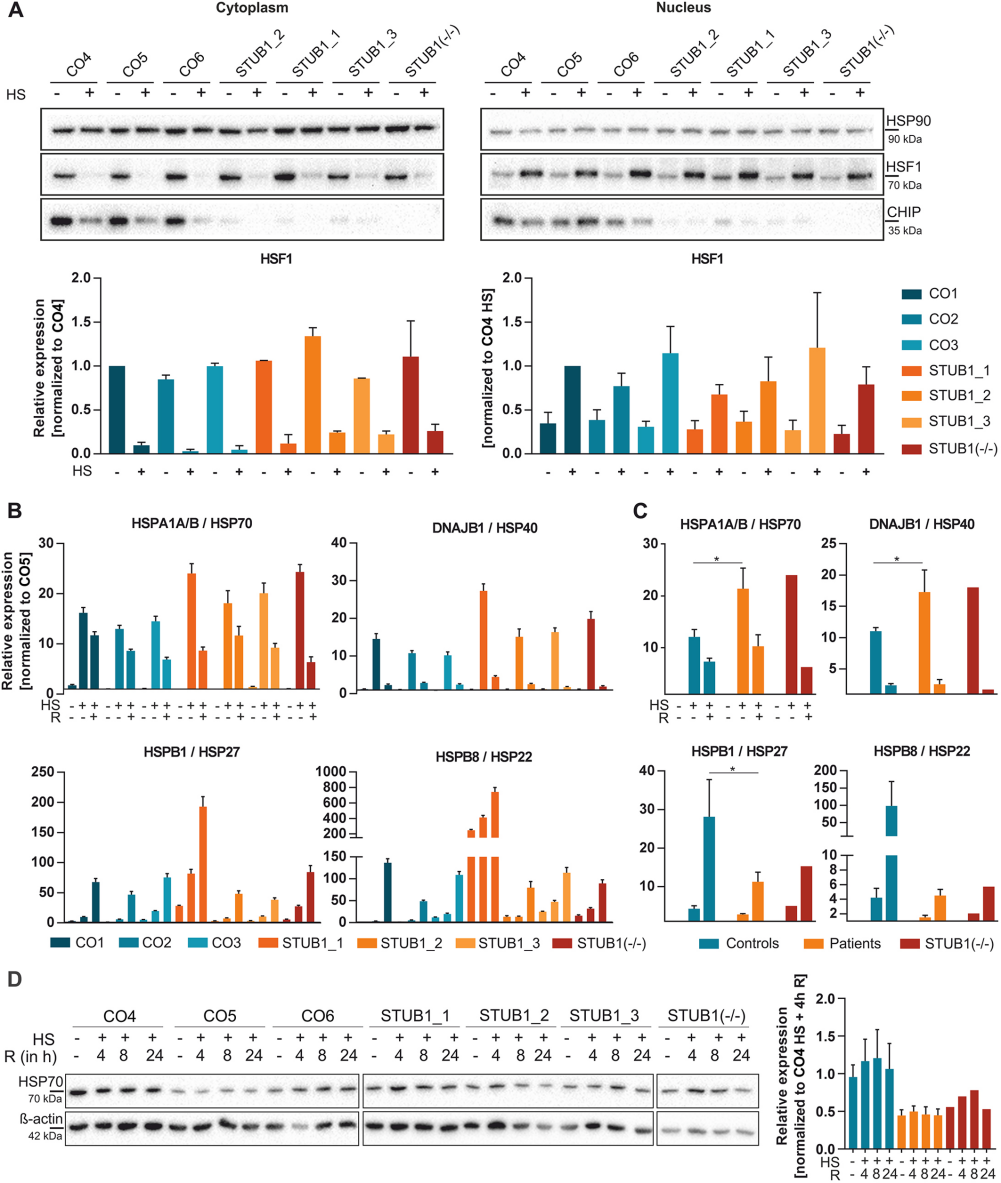
**Mutant CHIP does not impair the HSR in iPSC-derived CNs.** (A) Cytoplasmic and nuclear fractions of HSP90, HSF1 and CHIP before and after heat shock (heat shock, 1 h, 42.5°C) in CNs of three controls, three patients and STUB1(−/−). HSP90 and HSF1 levels were quantified densitometrically and normalized to HSP90, and to CO5/CO5 heat shock (HS). (B) Transcript analysis of *HSPA1A/B*, *DNAJB1*, *HSPB1* and *HSPB8* was performed by qRT-PCR. Values are normalized to CO5 and the housekeeping genes *GAPDH* and *TBP*. Each bar represents a triplicate. Data are mean±s.e.m. (C) Fold change of HSPs compared to baseline. Transcript levels of B were pooled for controls and patients. (D) Western blot analysis of HSP70 levels of unstressed and heat-shocked CNs with various times of recovery (R, in hours). HSP70 levels were quantified densitometrically, normalized to ß-actin and pooled for controls and patients. *n*=3 western blots were performed as technical replicates. Data are mean±s.e.m. *P<0.05; one-way ANOVA.

We next investigated the HSR in CNs after 1 h of heat shock at 42.5°C. To assess nuclear translocation of HSF1, we performed subcellular fractionation of the nucleus and cytoplasm. Proper separation of nuclei from the cytoplasmic fraction was verified by the presence and absence of histone 3 (H3), respectively (Fig. S2A). In unstressed conditions, HSF1 was highly expressed in the cytosol and weakly expressed in the nucleus. Upon heat shock, HSF1 was hyperphosphorylated, as seen by a mass shift, and was translocated to the nucleus (Fig. 5A), as almost no HSF1 remained in the cytosol after heat shock. HSF1 translocation and expression levels were not altered by mutant *STUB1* (Fig. 5A). CHIP levels were shown to diminish after heat shock in the cytosol in controls but did not increase drastically in the nucleus.

In terms of transcript levels, heat strongly induced the transcription of *HSPA1A/B* (encoding HSP70) and *DNAJB1* (encoding HSP40) (Fig. 5B,C). This induction was significantly higher in patients and STUB1(−/−) compared to controls [mean±s.e.m.; *HSPA1A/B* in controls, 12.06±1.18-fold; in patients, 21.38±3.25-fold (*P*=0.017); and in STUB1(−/−), 24-fold (*P*=0.03). *DNAJB1* in controls, 11.06±0.46 fold; in patients, 17.28±2.9-fold (*P*=0.038); and in STUB1(−/−), 18-fold (*P*=0.1)]. After 4 h of recovery, levels decreased in all lines again [*HSPA1A/B* in controls, 7.31±0.54-fold; in patients, 10.26±1.81-fold; and in STUB1(−/−), 6.3-fold. *DNAJB1* in controls, 2.40±0.22-fold; in patients, 2.58±0.61-fold, and in STUB1(−/−): 1.7-fold]. *HSPB1* (encoding HSP27) and *HSPB8* (encoding HSP22) transcription slightly increased upon heat shock by two to fourfold but highly increased after 4 h recovery at 37°C [*HSPB1* in controls, 28.16±7.86-fold; in patients, 11.25±2.05-fold (*P*=0.037); and in STUB1(−/−), 15.3-fold (*P*=0.3). *HSPB8* in controls, 98.5±57.65-fold; in patients, 4.5±0.7-fold (*P*=0.11); and in STUB1(−/−), 5.7-fold (*P*=0.3)]. The increase of *HSPB1* was significantly higher in controls compared to patients. However, variability for *HSPB1* and *HSPB8* was very high between lines, probably because of low basal expression levels. *HSP90AA1* (HSP90), *HSPA8* (HSC70) and *HSPA5* (BiP/GRP78) were neither strongly altered upon heat shock or recovery nor different between patients and controls (Fig. S2B,C).

Unexpectedly, in terms of protein levels, HSP70 expression under basal conditions was already very high in CNs, with slight variations between lines and independent of *STUB1* mutations (Fig. S2D). We barely saw an increase of HSP70 after heat shock and at various times of recovery [increase after 1 h heat shock plus 4 h recovery normalized to baseline (mean±s.e.m.) of controls, 1.27±0.3-fold; patients, 1.12±0.15-fold; STUB1(−/−), 1.25-fold]. However, when absolute HSP70 levels were compared, we saw slightly lower levels in patients compared to controls [unstressed HSP70 level of controls, 1±0.16; patients, 0.47±0.07 (*P*=0.27); and STUB1(−/−), 0.58. Heat shock (1 h) plus 4 h recovery HSP70 level of controls, 1.22±0.29; patients, 0.52±0.07 (*P*=0.25); and STUB1(−/−), 0.73] (Fig. 5D).

In summary, we were able to show that HSF1 translocates to the nucleus upon heat shock in CNs and that this leads to the induction of *HSPA1A/B* and *DNAJB1* transcripts, which surprisingly did not translate to increased HSP70 protein levels. Induction of *HSPA1A/B* and *DNAJB1* was significantly higher in patients and STUB1(−/−) compared to controls. Furthermore, we barely saw an induction of HSP70 on protein level in both controls and patients.

### Proteome analysis of CNs reveals impaired protein folding and ubiquitination in SCAR16 patients

**Fig. 6.**
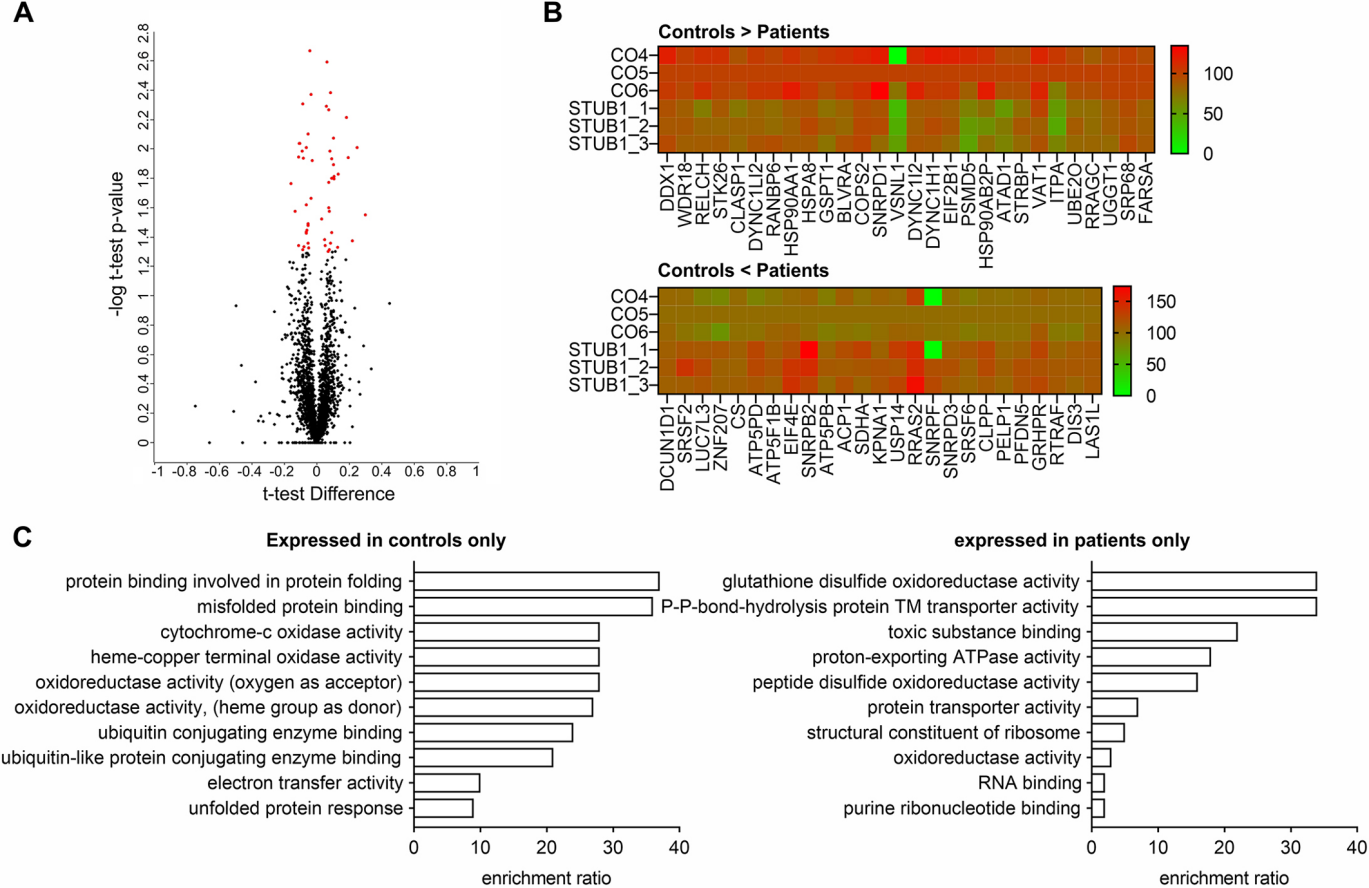
**Proteome analysis of CNs from three controls and three STUB1 patients revealed protein (re)folding disturbances and increased oxidative stress in patients.** (A) The volcano plot illustrates significantly differentially expressed proteins in patients versus controls. The –log 10 two-tailed unpaired Student's *t*-test *P*-value is plotted against the *t*-test difference in terms of controls versus patients. The significance threshold is set to *P*=0.05/1.30 (−log 10). (B) Heatmap displaying significantly dysregulated proteins, for proteins downregulated (upper panel) and upregulated in patients (lower panel). Values are given as log 10 LFQ intensity. Control line CO5 is set to 100%. (C) GO analysis with WebGestalt of proteins expressed in at least two of three controls but not in patients (left panel), and in at least two of three patients but not in controls (right panel). Proteins were subjected to GO classification in terms of molecular function. A threshold of five proteins per classification was set. Enrichment ratios of GO terms are shown.

Proteomic analysis based on mass spectrometry (MS) with liquid chromatography (LC)/MS-MS and label-free quantification (LFQ) was applied to identify quantitative differences in proteome-wide protein levels between CNs of controls and patients (*n*=3 for both biological groups). We identified 53 proteins with significantly altered levels (Fig. 6A); 28 proteins were increased (Fig. 6B, upper panel) and 25 proteins were reduced in controls compared to patients (Fig. 6B, lower panel). Additionally, 185 proteins were exclusively expressed in at least two control cell lines but not in any patient cell line (Table S3), and 228 proteins were exclusively expressed in at least two patient cell lines but not in any control cell line (Table S4). Based on WebGestalt analysis (Liao et al., 2019) of proteins that were exclusively expressed in at least two controls, gene ontology (GO) terms associated with protein folding and refolding, and the ubiquitin system, were highly enriched (Fig. 6C, left panel). In contrast, GO terms associated with oxidative stress coping were enriched in patients only (Fig. 6C, right panel). In terms of specific interaction partners of CHIP, we found differences between controls and patients for some proteins, including PSEN1/2, IGF1R, TRAF2 and TRAF6 (Fig. S3).

## DISCUSSION

Proteotoxic stress and a compromised HSR are associated with many neurodegenerative diseases, such as Alzheimer's disease, Parkinson's disease, Huntington's disease and amyotrophic lateral sclerosis (reviewed by San Gil et al., 2017). This suggests an innate susceptibility of neurons to stress, homeostatic changes and disturbances in protein quality control, thereby highlighting the significance of chaperones in neuroprotection.

In this study, we investigated the pathological effect of *STUB1* mutations causing SCAR16 on the HSR. Analyzing patient-derived fibroblasts, we confirmed an impaired HSR induction on transcript level and an impaired HSR recovery on protein level. Furthermore, we developed a human iPSC-based SCAR16 disease model with CNs as the affected cell type. Despite differences in heat-inducible transcript levels in *STUB1*-mutant CNs, we could not detect an impaired heat shock induction or recovery at the protein level in CNs of *STUB1* patients.

In a rodent model of SCAR16, CHIP^−/−^ mice exhibit decreased stress tolerance, pronounced heat sensitivity, increased oxidative stress and lethality upon heat shock (Dai et al., 2003; Min et al., 2008). *In vitro*, CHIP knockdown in mesodermal cell lines caused reduced cell viability upon prolonged heat stress and impaired induction and recovery of the HSR (Dai et al., 2003; Kim et al., 2005; Qian et al., 2006; Zhang et al., 2015).

In human SCAR16 cell models, we found a striking difference in heat stress tolerance between cell types. Although fewer than 30% of fibroblasts survived 4 h of heat shock at 42.5°C, more than 95% of neurons were still viable. This might indicate a higher stress vulnerability of fibroblasts and a stronger response to this toxic stimulus. Although Dai et al. (2003) reported 50% lethality of murine CHIP(−/−) fibroblasts after 60 min at 42°C, our patient-derived fibroblasts did not show relevant lethality after 60 min of heat shock at 42.5°C. This difference might be due to low levels of remaining CHIP in patient fibroblasts with residual activity that potentially rescues the drastic toxic effect seen in CHIP knockout fibroblasts. The better stress coping of CNs could be a result of the immaturity of the CNs as efficiency of the proteostasis network strongly declines with terminal differentiation (Vonk et al., 2020) and with aging (Morishima et al., 2008).

**Fig. 7.**
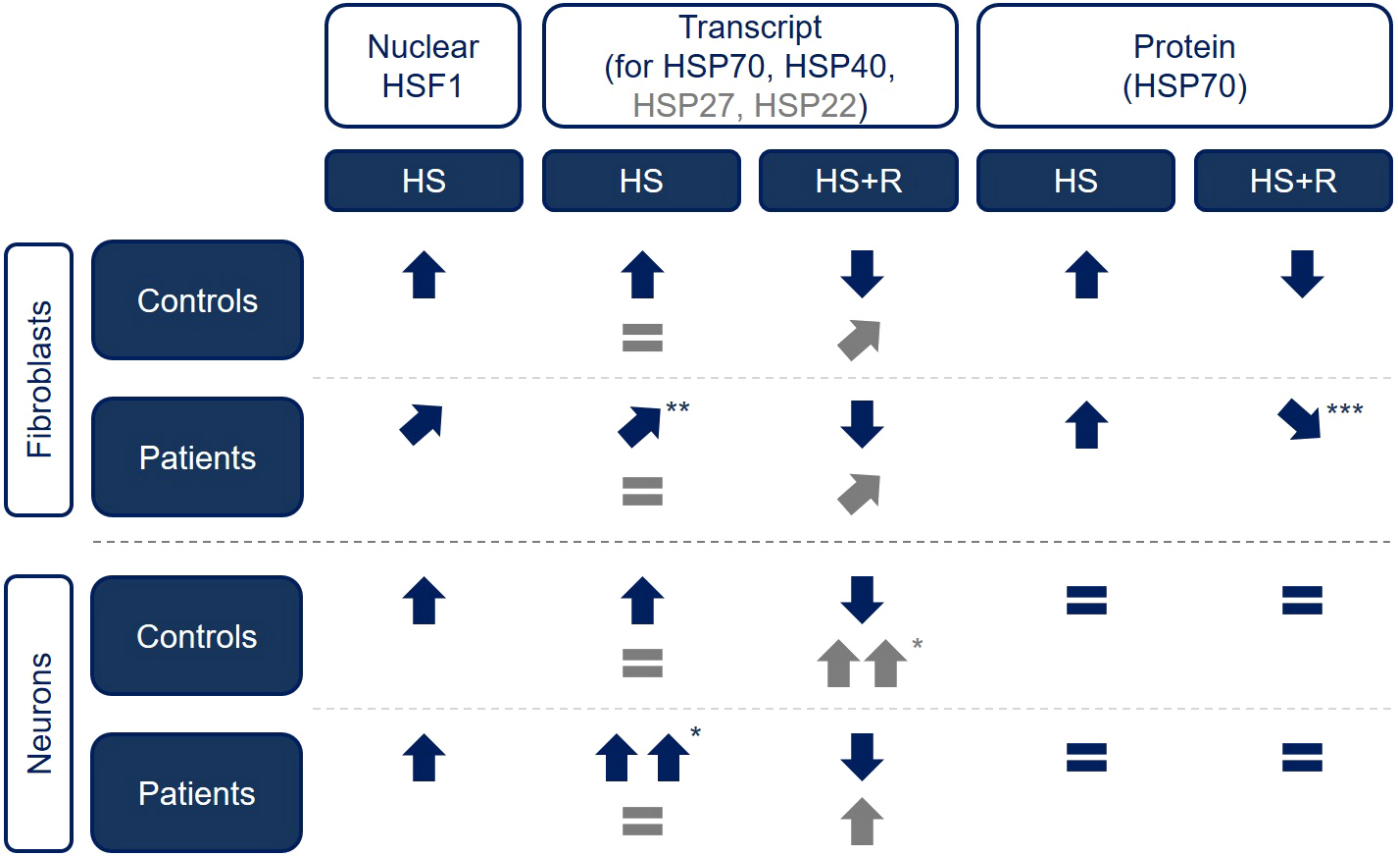
**Comparative overview of heat shock-related changes in control and SCAR16 patient fibroblasts and CNs.** Experimental results of control- and patient-derived fibroblasts (Fig. 2) and control- and patient iPSC-derived CNs (Fig. 5) are summarized. *P<0.05, **P<0.01, ***P<0.001. **↓**, reduction of level; ↑, increase of level; **=** no change in level. Comparison is based on unstressed condition for heat shock, and on heat shock for heat shock and recovery. HS, heat shock; HS+R, heat shock and recovery.

Our data imply that *STUB1* mutations, and thereby dysfunctional CHIP, do not affect the cell viability negatively upon heat shock in fibroblasts and CNs. We next evaluated the induction of HSR and its recovery in patient- and healthy control-derived fibroblasts. Using immunocytochemical analysis of heat shocked cells, we showed that HSF1 translocated to the nucleus in patients and controls, with a trend towards lower levels in controls. Furthermore, when different transcript levels in fibroblasts were analyzed, we observed a lower increase in *HSPA1A/B* and *DNAJB1* transcript levels upon heat shock in patients compared to controls. This might be caused by slightly lower nuclear HSF1 levels and/or by differential post-translational modifications (PTMs) of HSF1, as PTMs were reported to strongly alter the activity of HSF1 (reviewed by Gomez-Pastor et al., 2018). Surprisingly, at the protein level, we did not see reduced HSP70 levels in patients compared to controls after a 4 h recovery from heat shock as would be expected from the lower transcript levels, but instead we observed a six to sevenfold increase compared to unstressed levels in both biological groups. Yet, we see an impaired HSR recovery, as indicated by the remaining high levels of HSP70 after prolonged recovery (8 h and 24 h), in line with results presented by Qian et al. (2006). This leads to the conclusion that either HSP70 ubiquitin tagging is reduced in CHIP-mutant patient cells or that the amount of misfolded proteins in patients is simply higher, requiring a prolonged high expression of HSPs in those cells, or a combination of both. The maintained HSP70 protein expression in SCAR16 patient-derived cells might also lead to the observed similar cell viability upon prolonged heat shock. In conclusion, we confirm an impaired induction and recovery of the HSR caused by dysfunctional CHIP, which is the first such observation in patient-derived cells (summarized in Fig. 7).

As SCAR16 primarily causes degeneration of the central nervous system, we next sought to analyze the HSR in patient neurons. We therefore generated patient-specific iPSCs from fibroblasts and differentiated those, together with gender-matched controls, into CNs as a disease-relevant cell type. This provides a disease model with endogenous protein levels and the patient's own genetic background. Although mutations in STUB1_1 and STUB1_3 do not alter *STUB1* transcript levels, protein stability is strongly reduced by the mutations, leading to a lower CHIP level (Heimdal et al., 2014; Ronnebaum et al., 2014; Pakdaman et al., 2017; Kanack et al., 2018; Shi et al., 2018; Madrigal et al., 2019). STUB1_2 shows reduced transcript and protein levels.

Analyzing the HSR, we could show that the translocation of HSF1 from the cytoplasm to the nucleus upon heat shock occurs invariable of CHIP functionality, suggesting no direct impact of CHIP mutations on trimerization of HSF1 or its translocation in CNs. CHIP levels declined upon heat shock in the cytoplasm, but did not correspondingly increase in the nucleus. Potentially, nuclear CHIP levels did already return to baseline in our experimental setup with 1 h heat shock, as Anderson and colleagues showed a strong accumulation in the nucleus only 5-10 min after heat shock and a decrease back to baseline within 30-60 mins (Anderson et al., 2010).

When transcript levels of HSPs were analyzed, we observed a higher increase in *HSPA1A/B* and *DNAJB1* upon heat shock in patient neurons compared to controls, independent of nuclear HSF1 levels. This might be caused by differential PTMs of HSF1 as described above. Higher *HSPA1A/B* and *DNAJB1* transcription might also be caused by ‘preconditioning’ to stress and thermotolerance in patients: levels of misfolded proteins are presumably higher in CHIP mutant cells, causing mild proteotoxic stress and an induction of the HSR before the heat stimulus which potentiates the HSR upon subsequent stress exposure (Burdon, 1987). Stress preconditioning might only play a role in CNs and not in fibroblasts as non-dividing cells are more vulnerable to proteotoxic stress in general.

Transcript levels of the sHSPs *HSPB1* (coding for HSP27) and *HSPB8* (coding for HSP22) were strongly increased during the recovery steps from heat shock, indicating two stages of transcript induction with an early induction of *HSPA1A/B* and *DNAJB1* and, unexpectedly, a delayed induction of *HSPB1* and *HSPB8*. HSP27 and HSP22 bind to unfolded proteins, prevent their aggregation and protect cells from toxicity caused by aggregates. Both sHSPs also enable stress resistance and inhibit apoptosis (Acunzo et al., 2012). This might explain the higher levels in controls compared to patients and delayed induction. Interestingly, mutations in both *HSPB1* and *HSPB8* induce neurodegeneration, highlighting the importance of both sHSPs in the stress response in neurons in particular.

Unexpectedly, at the protein level, we neither detected a prominent induction of HSP70 protein nor a difference in HSP70 level upon recovery. HSP70 levels at 4 h recovery after heat stress were only 1.1- to 1.2-fold higher than at its basal levels, compared to six to sevenfold higher levels in fibroblasts. These differences cannot be explained by varying CHIP expression in these cell types, as the basal CHIP protein expression levels in fibroblasts and CNs were comparable (Fig. S4), but there might be several other reasons: (1) an impaired HSR in the brain might be relevant and disease-causing but not in neurons; (2) alterations of the HSR that were seen in patient-derived fibroblasts but not in CNs are not disease-relevant, as the diseased cell type does not show any changes; or (3) the low HSR is an *in vitro* artifact of the monoculture and the culture conditions, as the basal HSP70 level is already very high. As persistently high levels of HSP70 are detrimental to cells (Feder et al., 1992; Volloch and Sherman, 1999), an additional induction of HSP70 upon stress might be prevented in iPSC-derived CNs. Considering the role of CHIP in this process, we observed only slightly lower HSP70 protein levels in patients but no difference in fold changes. Furthermore, the comparison of STUB1(−/−) with its isogenic control revealed very similar results to the comparison of patients and controls. This confirms our findings in patient lines and supports the limited effect of CHIP on the HSR in CNs (summarized in Fig. 7).

Although the HSR is evolutionarily well conserved, tissue and cell variability of the chaperone in humans is prominent: Hageman and Kampinga (2009) analyzed the temporal and the spatial expression pattern of 45 tissues using the UniGene database and observed strong tissue specificity for the expression of HSPH, HSPA and DNAJ isoforms. Notably, the expression of *HSPA1A* is highly variable between tissues and developmental stages (embryoid body to adult). Focusing on the brain, histological analyses after heat shock in rodents showed the strongest HSP70 induction in the dentate gyrus, hypothalamus and cerebellum (Blake et al., 1990; Li et al., 1992). Of interest, a comparison of HSR in neurons and glia *in vitro* revealed a higher induction of HSP70 in glial cells compared to neurons (Nishimura et al., 1991; Marcuccilli et al., 1996; Vogel et al., 1997; Batulan et al., 2003). This might be caused by differential co-chaperone levels: HSPBP1 expression in neurons is higher than in astrocytes and is directly linked to HSR inhibition (Zhao et al., 2017); and HSPB1/HSP27 levels are oppositely correlated (Satoh and Kim, 1995). However, although HSP27 levels do not increase upon heat shock in astrocytes, this increase is observed in neurons (Satoh and Kim, 1995). Other HSPs, such as DNAJB2A (HSP70 family) and HSJ1a/b (HSP40 family), were reported to be highly enriched in neuronal tissue (Cheetham et al., 1992; Chapple and Cheetham, 2003). In our study, we additionally observed a strong cell specificity of *HSPB1* and *HSPB8*, with higher expression under basal conditions in fibroblasts, but higher induction upon recovery of heat stress in neurons. This might point towards an important role for both sHSPs in stress coping in CNs.

For a broader picture, proteomic analysis of control and patient CNs was performed. Comparisons point towards aberrations in protein folding and the ubiquitin system, as well as an increased oxidative stress level in unstressed patient cells. This is in line with reports that show that mutations alter the ability of CHIP to ubiquitinate its substrates (Heimdal et al., 2014; Ronnebaum et al., 2014; Pakdaman et al., 2017; Kanack et al., 2018; Shi et al., 2018). However, with more than 200 E3 ligases encoded in the human body, substrates are not exclusively ubiquitinated by one E3 ligase; for example, parkin and CHIP act redundantly on some substrates (Morishima et al., 2008). Furthermore, keeping in mind the function of CHIP in tagging misfolded proteins with ubiquitin for degradation, the importance of CHIP might only show up in a state with a high abundancy of misfolded proteins.

In summary, our results question the role of impaired HSR in SCAR16 neuropathology and point to protein degradation and oxidative stress as potentially more critical factors in the pathogenesis of CHIP-related neurodegeneration. By demonstrating major differences in the HSR of fibroblasts and induced CNs of SCAR16 patients, this study highlights the need for the careful selection of proper cell types for modeling human diseases.

## MATERIALS AND METHODS

### Patients

Three patients with genetically confirmed compound heterozygous or homozygous mutations in *STUB1* were included in this study. Patient STUB1_1 had a homozygous c.367C>G; p.L123V mutation, whereas patients STUB1_2 and STUB1_3 carried the compound heterozygous mutations c.355C>T; p.R119*; c.880A>T, p.I294T and c.433A>C, p.K145Q; c.728C>T, p.P243L, respectively (Hayer et al., 2017). All patients were severely affected by SCAR16 with ataxia (3/3), spasticity (3/3), epilepsy (2/3), dementia (2/3) and hypogonadism (1/3). The controls were age- and gender-matched for fibroblast analyses and gender-matched for iPSC-derived neuronal analyses. Detailed information on study participants and cell lines is provided in Table S1. The study was approved by the Ethics Committee of the University of Tübingen (vote 598/2011BO1). Informed written consent was obtained from all participants or their legal guardian.

### Fibroblast cultivation

Human dermal fibroblasts were obtained from skin biopsies and maintained at 37°C, 5% CO_2_ and 100% relative humidity in fibroblast medium consisting of Dulbecco's modified eagle medium (DMEM) (Merck Millipore) supplemented with 10% fetal bovine serum (FBS) (Thermo Fisher Scientific) in cell culture flasks. Upon reaching high confluency, cells were split by washing with PBS followed by 5 min trypsination and passaging into new flasks, or seeding at a defined density depending on the assay (20,000 cells/cm^2^ for immunocytochemical analysis and 30,000 cells/cm^2^ for protein analysis).

### Reprogramming of fibroblasts to iPSCs

The generation of iPSCs from fibroblasts was performed according to a previously published protocol (Okita et al., 2011) with minor modifications. In brief, 10^5^ human dermal fibroblasts were nucleofected with 1 µg of each episomal plasmid [pCXLE-hUL, pCXLE-hSK and pCXLE-hOCT4 (Addgene numbers 27080, 27078 and 27076, respectively)] with Nucleofector 2b (Lonza). Fibroblasts were cultivated in fibroblast medium before adding 2 ng/ml FGF2 (Peprotech) on day 2. The following day, medium was changed to Essential 8 (E8) medium [DMEM/F12 (Life Technologies), 64 mg/l L-ascorbic acid 2-phosphate magnesium (Sigma-Aldrich), 1% insulin-transferrin-selenium-supplement (100×) (Life Technologies), 10 ng/ml FGF2, 2 ng/ml TGFß1 (Peprotech) and 100 ng/ml heparin (Sigma Aldrich)] with 100 μM NaB (Sigma-Aldrich). After 3 to 4 weeks, with a change of medium every other day, colonies were manually picked and expanded onto Matrigel-coated plates (Corning) in a feeder-free system in E8 medium. The iPSCs were frozen in E8 medium with 40% KnockOut serum replacement (Life Technologies), 10% dimethyl sulfoxide (Sigma-Aldrich) and 10 μM Y-27632 (Selleckchem). Genomic integrity was confirmed by excluding plasmid integration and performing whole-genome single nucleotide polymorphism genotyping [Infinium OmniExpressExome-8-BeadChip (Illumina) or CytoScan HD technology (Affymetrix), and copy number analysis (CNVPartition plug-in (Illumina)], and resequencing of the mutation sites. Pluripotency was confirmed by ALP expression, immunocytochemical analysis of pluripotency markers, transcript analysis of pluripotency genes and embryoid-body-based differentiation of iPSCs into cells of all three germ layers. For detailed descriptions see Schuster et al. (2018).

### Targeted *STUB1* knockout with CRISPR/Cas9

To generate the *STUB1* knockout line STUB1(−/−), we targeted exons 2 and 3 of *STUB1* in a dual-cleavage approach to enhance the efficiency of knockout generation. CRISPR-RNAs (crRNAs) (Integrated DNA Technologies) were designed using the CRISPOR web tool (Haeussler et al., 2016). The iPSCs of control line CO5 were nucleofected with two crRNA-ATTO550-tracrRNA (Integrated DNA Technologies) ribonucleoprotein complexes with Cas9 protein (Integrated DNA Technologies), followed by fluorescence-activated cell sorting of ATTO550^+^ cells with a Sony Cell Sorter SH800Z, single cell seeding and manual picking and expansion of clones. Homozygous or heterozygous knockout was validated by PCR analysis and Sanger sequencing. The top five exonic off-target effects, as predicted by CRISPOR for each cleavage site, were excluded by Sanger sequencing in the isogenic control, as well as the generated clones. For detailed experimental setup and characterization of STUB1(−/−) see Schuster et al. (2019).

### Neuronal differentiation

To model SCAR16 *in vitro*, we generated neurons of cortical layer V and VI according to a published protocol (Shi et al., 2012; Rehbach et al., 2019) with modifications. In brief, iPSCs were dissociated with 0.02% EDTA (Carl Roth) in PBS (Sigma-Aldrich) and seeded at a density of 3×10^5^/cm^2^ in E8 medium supplemented with 10 µM Y-27632. The following day, the medium was replaced by neural induction medium [1:1 N2/B27, 500 nM LDN-193189 (Th. Geyer) and 10 µM SB431542 (Sigma-Aldrich)], which, from there onwards, was changed every day. The neural induction medium was supplemented with 20 ng/ml FGF2 on day 8. The next day, cultures were split by detachment with Accutase (Sigma-Aldrich) for 10 min and seeded in N2/B27 medium with 20 ng/ml FGF2 and 10 µM Y-27632 onto Matrigel-coated six-well plates. N2/B27 medium with 20 ng/ml FGF2 was added on the next day. From day 11 onward, cells were cultured in N2/B27 medium with a change of medium every other day. Neural precursors were dissociated and frozen on day 19. For maturation, frozen precursors were thawed and seeded in N2/B27 medium with 10 µM Y-27632, with a change of medium every other day. On day 26, cells were detached with Accutase and reseeded at the desired assay density (for immunocytochemical staining, 1×10^5^/cm^2^; and for protein and RNA isolation, 4-6×10^5^/cm^2^) on poly-l-ornithine (Sigma-Aldrich) and Matrigel-coated wells. On day 27 and 29, the medium was changed to N2/B27 supplemented with 10 µM PD0325901 (Tocris) and 10 µM DAPT (Sigma-Aldrich). From day 31 and up to the analysis on day 36, the cells were cultured in N2/B27 medium with medium changes every other day. For heat shock experiments, cells were exposed to 42.5°C with prewarmed medium for 1 h, with direct harvesting of cells for RNA isolation and protein fractionation, and an additional 4, 8 or 24 h recovery at 37°C for protein analysis.

### Immunocytochemistry and image analysis

Cells were washed with PBS followed by fixation with 4% paraformaldehyde (Merck Millipore) and subsequent washing. Fixed cells were permeabilized and blocked with 5% bovine serum albumin (Sigma-Aldrich) in PBS with 0.1% Triton X-100 (Carl Roth). Afterwards, cells were stained with anti-β-III-tubulin (TUJ, mouse, 1:1000, T8660, Sigma-Aldrich), anti-CTIP2 (rat, 1:200, ab18465, Abcam), anti-HSF1 (rabbit, 1:500, 4356T, Cell Signaling Technology) and/or anti-HSP70 (mouse, 1:500, ADI-SPA-810, Enzo Life Sciences), all followed by labeling with Alexa Fluor-conjugated secondary antibodies (Invitrogen). Hoechst 33258 staining (1:10,000, H1398, Invitrogen) was used to counterstain for nuclei. Coverslips were mounted with Dako mounting solution (Agilent Dako) onto microscope slides. Four or five random fields per coverslip per cell line were used for quantification. Images were acquired using an Observer Z1 fluorescence microscope (Zeiss), and exposure time was kept constant. Quantifications were conducted using the cell counter plug-in in FiJi (Schindelin et al., 2012) and threshold mask settings in ImageJ (https://imagej.nih.gov/ij/).

### Transcript analysis by qRT-PCR

For RNA isolation, cells were scraped off in RLT buffer (Qiagen) and lysed in the well. RNA was isolated with an RNeasy Mini Kit (Qiagen) according to the manufacturer's instructions. A total of 500 ng RNA was reverse-transcribed into cDNA using a RevertAid First Strand cDNA Synthesis Kit (Thermo Fisher Scientific) according to the manufacturer's instructions. For real-time PCR, 3 µl of 1.25 ng/µl cDNA was mixed with 2 µl of 2 µM primer pairs and 5 µl of SYBR Green Select Master Mix (Applied Biosystems). Primers are listed in Table S2. The qRT-PCR program was as follows: 50°C for 2 min, 95°C for 2 min, followed by 40 cycles of 95°C for 1 s, 60°C for 30 s and 72°C for 5 s, and subsequently 95°C for 15 s, 60°C for 1 min and 95°C for 15 s. The specificity of PCR products was confirmed by melting curve analysis. Real-time PCR amplifications were performed on a Viia7 Real-Time PCR System (Applied Biosystems) and primers were run in triplicate. The housekeeping genes GAPDH and TBP were amplified to standardize the amount of sample cDNA. Analysis was performed with QuantStudio Software V1.3 (Thermo Fisher Scientific).

### Cell viability analysis

Fibroblasts were seeded at a density of 12.5×10^4^/cm^2^ on 96-well plates in triplicate. CNs were seeded at a density of 3×10^5^/cm^2^ on D33 on 96-well plates coated with poly-l-ornithine and Matrigel in quadruplicate. To assess cell viability upon heat shock, the CyQuant direct cell proliferation assay (Thermo Fisher Scientific) was used according to the manufacturer's instructions. In brief, CQD nucleic acid stain and CQD background suppressor dye were diluted in PBS and added to the cells, followed by 1 h incubation at 37°C. Triton X-100 (1%) was used as a negative control for cell viability. The plate reader SpectraMax M (Molecular Devices) was set to 42.5°C or 44°C, and plates were measured for 6 h with excitation/emission at 485/525 nm.

### Protein isolation

Pellets of primary fibroblasts or CNs were lysed in RIPA buffer (Sigma-Aldrich) containing 1×cOmplete protease inhibitor cocktail (PI) (Roche) for 45 min on a rotator at 4°C. Cell debris was pelleted at 15,800 ***g*** at 4°C for 30 min. Protein concentration was determined using the Pierce BCA Protein Assay Kit (Thermo Fisher Scientific) according to the manufacturer's instructions.

### Subcellular fractionation

To separate nuclei from cytoplasm in CNs, an Abcam protocol was applied with slight modifications. In brief, cells were scraped off on ice in fractionation buffer [20 mM HEPES (pH 7.4) (Carl Roth), 10 mM KCl (Carl Roth), 2 mM MgCl_2_ (Merck Millipore), 1 mM EDTA (Carl Roth), 1 mM EGTA (AppliChem), 1 mM dithiothreitol (AppliChem) and 1×PI] and incubated for 15 min on ice. Samples were then passed through a 27-gauge needle (Sigma-Aldrich) ten times, then incubated on ice for 20 min. Samples were centrifuged at 750 ***g*** for 5 min at 4°C and supernatant containing cytoplasm was transferred to a fresh tube. One quarter of the total amount of 5×RIPA buffer [750 mM NaCl (VWR), 5% Igepal CA-630 (Sigma-Aldrich), 2.5% Sodium deoxycholate (Carl Roth), 0.5% SDS (Sigma-Aldrich) and 250 mM Tris (pH 8.0) (AppliChem)] with 1×PI was added. The nuclear pellet was washed with fractionation buffer followed by ten more passes through a 27-gauge needle and 10 min centrifugation at 750 ***g*** at 4°C. The supernatant was discarded, the pellet was resuspended in 1×RIPA buffer plus PI and sonicated to shear genomic DNA. Protein isolation was performed according to the procedure described above.

### Western blotting and densitometric analysis

Protein (10-30 µg) was eluted in 5×pink buffer (Thermo Fisher Scientific) at 95°C. Samples were separated on 10% polyacrylamide gels in 1×NuPAGE MOPS SDS running buffer (Novex) and transferred onto a Hybond-P polyvinylidene difluoride membrane (Merck Millipore). Membranes were blocked in 5% milk in TBS-T, incubated overnight with primary antibodies [ß-actin (mouse, 1:20,000, A5441, Thermo Fisher Scientific), CHIP (rabbit, 1:10,000, ab134064, Abcam), H3 (horseradish peroxidase (HRP)-tagged, 1:100,000, ab21054, Abcam), HSF1 (rabbit, 1:1000, 4356T, Cell Signaling Technology), HSP70 (mouse, 1:50,000, ADI-SPA-810, Enzo Life Sciences), HSP90 (mouse, 1:100,000, ADI-SPA-831-050, Enzo Life Sciences)] in Western Blocking Reagent (Roche) at 4°C, followed by three washes with TBS-T and incubation with HRP-conjugated secondary antibodies (Jackson ImmunoResearch) for 1 h at room temperature. Proteins were visualized using the Immobilon Western chemiluminescent HRP substrate (Merck Millipore). Bands were quantified with ImageJ and normalized to respective loading controls.

### RNA Sequencing analysis

Isolated RNA at a concentration of 20 ng/µl was further purified with a TruSeq mRNA v2Kit (polyA, Illumina). Biological triplicates were sequenced on a HiSeq2500 using paired-end chemistry and 2×125 cycles. The sequence depth was ∼100 million reads per sample. Runs were performed by deCODE Genetics.

To assess the differentiation state of generated CNs, cross-platform comparisons with spatiotemporal data from the developing human brain of the BrainSpan atlas were performed by pairwise comparison of generated CNs with each BrainSpan atlas sample. By ranking gene expression for each pairwise comparison, rank difference values for all genes were used to calculate Spearman’s rank correlation coefficients. A Wilcoxon rank-sum test was applied if a category of interest (neocortex, subcortex, ganglionic eminence or cerebellum) had significantly higher Spearman’s correlation coefficients than the background of all paired correlations. –Log 10 *P*-values of significant differences of CNs and spatiotemporal brain data were shown in heat maps.

### Mass spectrometry

Equal amounts of samples were purified using SDS PAGE (Invitrogen). Coomassie blue-stained gel pieces were excised and in-gel digested using trypsin as described previously (Borchert et al., 2010). Extracted peptides were desalted using C18 StageTips and subjected to LC/MS-MS analysis, performed on an Easy nano-LC (Thermo Fisher Scientific) coupled to an LTQ Orbitrap Elite (Thermo Fisher Scientific) as described previously (Franz-Wachtel et al., 2012). Equal amounts of the peptide mixtures were injected onto the column in high-performance LC (HPLC) solvent A (0.1% formic acid) at a flow rate of 500 nl/min and subsequently eluted with a 127 min segmented gradient of 5–33-50-90% of HPLC solvent B (80% acetonitrile in 0.1% formic acid) at a flow rate of 200 nl/min. Using an Orbitrap Elite, the 15 most intense precursor ions were sequentially fragmented in each scan cycle. In all measurements, sequenced precursor masses were excluded from further selection for 60 s. The target values were 5000 charges for MS/MS fragmentation and 106 charges for the MS scan. CNs of three controls (CO4, CO5 and CO6) and three *STUB1* patients (STUB1_1, STUB1_2 and STUB1_3) were analyzed.

### MS data processing

The data were processed using MaxQuant software suite v.1.5.2.8 (Cox and Mann, 2008). The database search was performed with MaxQuant, using the Andromeda search engine (Cox et al., 2011). MS/MS spectra were searched against a target-decoy Uniprot database consisting of 95,972 protein entries from *Homo sapiens* and 245 commonly observed contaminants. Full specificity was required for trypsin; up to two missed cleavages were allowed. Carbamidomethylation of cysteine was set as a fixed modification and oxidation of methionine and acetylation of the N-terminus were set as variable modifications. Initial mass tolerance was set to 4.5 ppm for precursor ions and 0.5 Da for fragment ions. Peptide, protein and modification site identifications were reported at a false discovery rate of 0.01, estimated by the target/decoy approach (Elias and Gygi, 2007). The label-free algorithm was enabled, as was the ‘match between runs’ option (Luber et al., 2010). LFQ protein intensities from the MaxQuant data output were used for relative protein quantification. Downstream bioinformatical analysis (two-sample *t*-tests and volcano plots) was performed using the Perseus software package, version 1.5.0.15. The data were filtered for contaminants, reverse and only identified by site entries.

### Statistical analysis

All statistical analyses were performed using GraphPad Prism software (version 8). One-way ANOVA or a two-tailed unpaired Student's *t*-test was applied. Statistical analysis was restricted to inter-biological comparison for the same experimental condition. *P*-values were corrected for multiple comparisons and *P*<0.05 was considered statistically significant. Unless indicated otherwise, all data are shown as mean±s.e.m.

## Supplementary Material

Supplementary information
