# Supplementary material for: CHIP mutations affect the heat shock response differently in human fibroblasts and iPSC-derived neurons
Source: Dis Model Mech. 2020 Oct 12;13(10):dmm045096. doi: 10.1242/dmm.045096 (PMC7578354; doi:10.1242/dmm.045096)
Supplement: Supplementary information [file dmm-13-045096-s1.pdf]

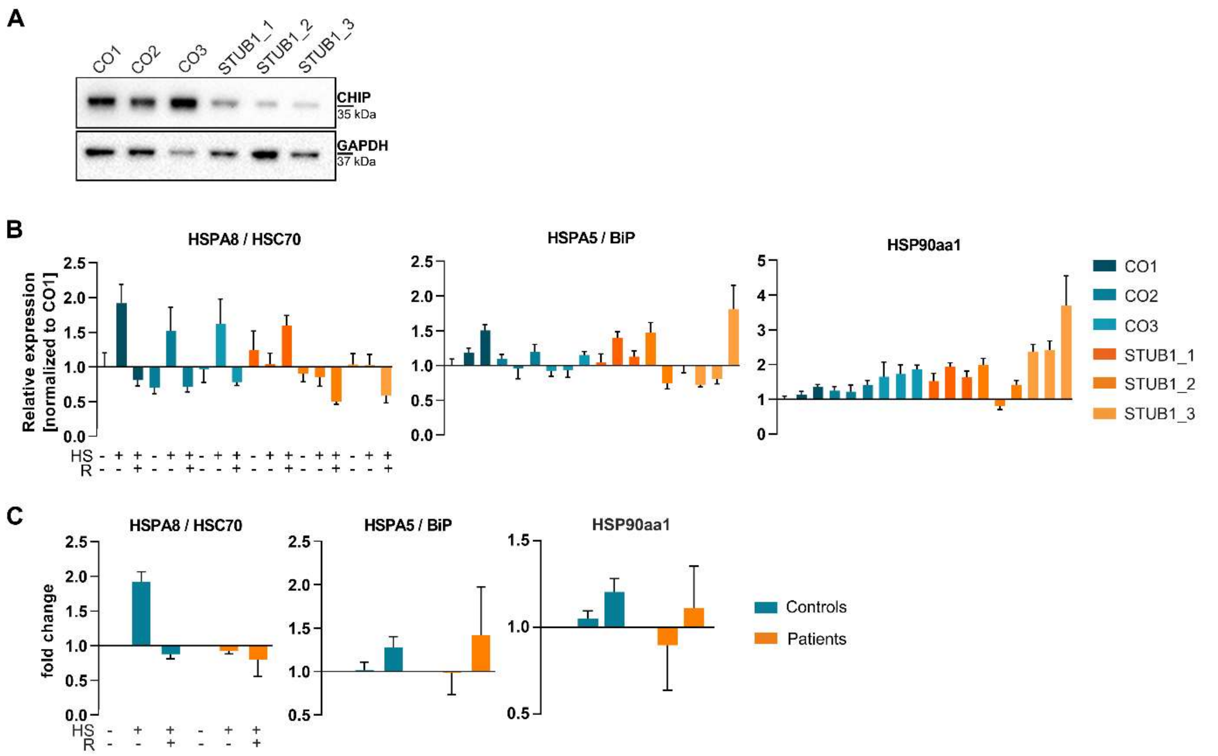

Fig. S1: Protein and transcript analysis of fibroblasts. (A) CHIP protein expression level was analyzed by Western Blotting, GAPDH is shown as housekeeping protein. One representative blot is shown. **(B)** Transcript analysis of *HSPA8, HSPA5 and HSP90aa1* was performed by qRT-PCR. Values are normalized to control line CO1 and the housekeeping genes *GAPDH* and *TBP*. Each bar represents a triplicate with mean ± s.e.m. **(C)** Fold change of HSPs compared to baseline. Transcript levels of (B) were pooled for controls and patients.HS: heat shock at 42.5°C for 1h. R: recovery at 37°C for 4h.

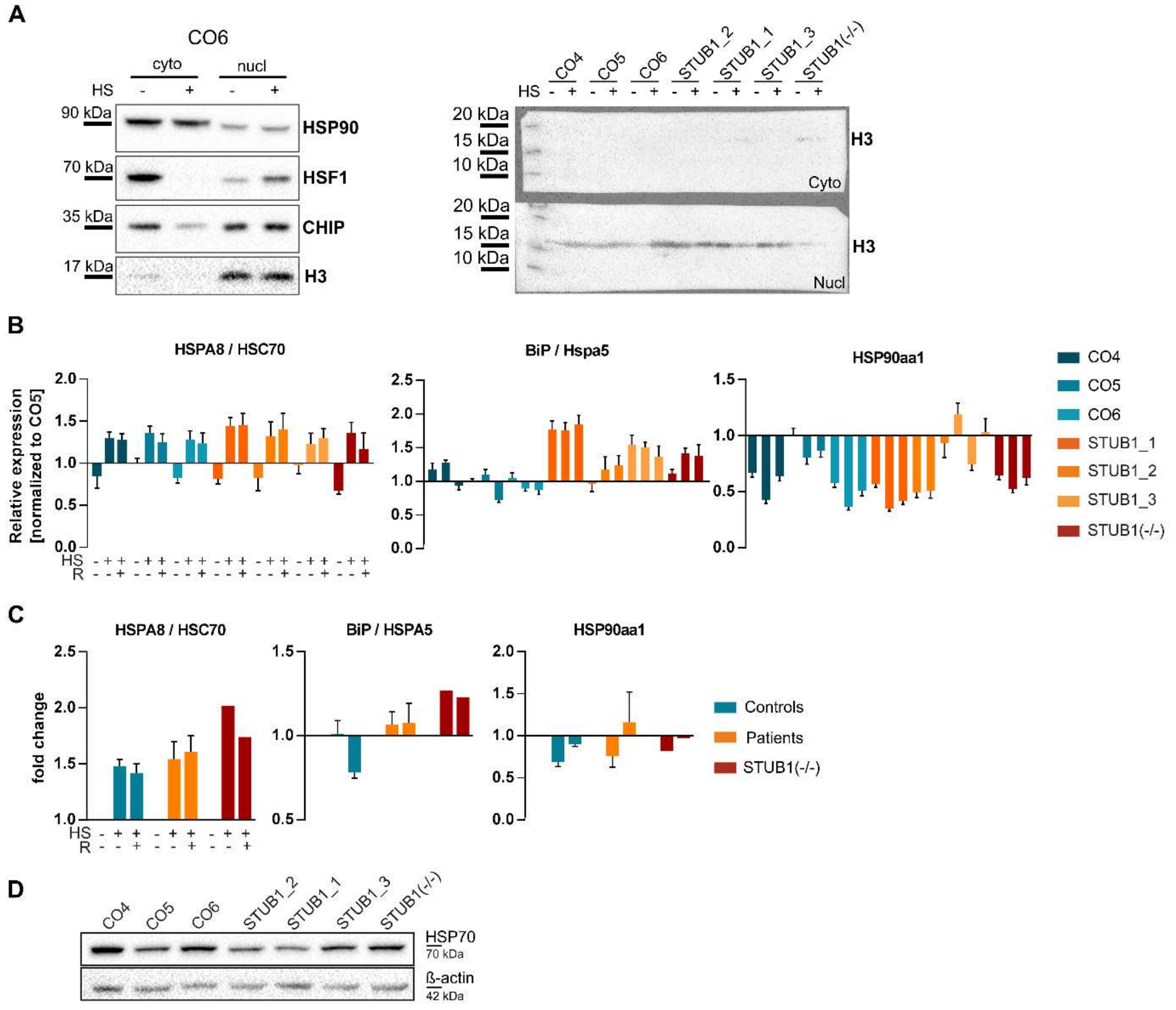

Fig. S2 | Mutant CHIP does not impair the HSR in iPSC-derived neurons. (A) Representative Western blot of cortical neurons derived from iPSC of control line CO6 demonstrating shift of HSF1 and HSP70 but not HSP90 from the cytoplasm to the nucleus upon heat shock (HS) of 42.5°C for 1h. Proper fractionation was verified for absence (cytosolic fraction) or presence (nuclear fraction) of histone 3 (H3) (right panel) for all 7 CN lines under all conditions. **(B)** Transcript analysis of *HSPA8, HSPA5 and HSP90aa1* was performed by qRT-PCR. Values are normalized to CO5 and the housekeeping genes *GAPDH* and *TBP*. Each bar represents a triplicate with mean ± s.e.m. HS: heat shock of 42.5°C for 1h. R: recovery at 37°C for 4h. **(C)** Fold change of HSPs compared to baseline. Transcript levels of (B) were pooled for controls and patients. **(D)** HSP70 protein expression was assessed in unstressed cells of 3 controls, 3 patients and STUB1(-/-). ß-actin was used as loading control. Cyto: cytoplasm; Nucl: nucleus.

Disease Models & Mechanisms • Supplementary information

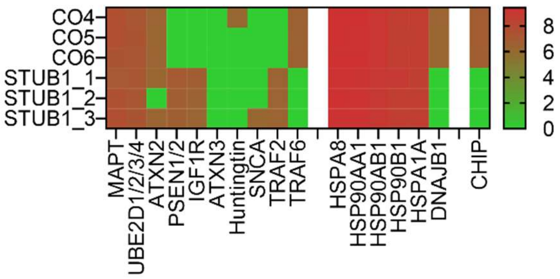

Fig. S3 | Expression of selected CHIP interaction partners by proteomic analysis of iPSC-derived neurons. Protein of cortical neurons of 3 Controls and 3 *STUB1* patients was analyzed by liquid chromatography-mass spectrometry/mass spectrometry (LC-MS/MS) and analyzed with Perseus. Values are given as –LOG10 label-free quantification (LFQ) values.

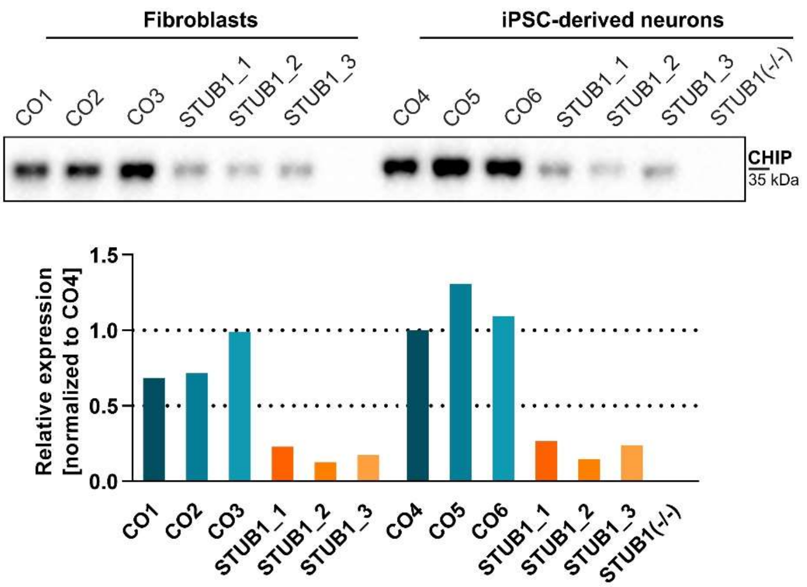

Fig. S4: Comparative CHIP protein analysis of fibroblasts and iPSC-derived neurons. (A) CHIP protein expression level was analyzed by Western Blotting. Bands are quantified densitometrically and normalized to total protein levels (Ponceau S) and CO4.

Disease Models & Mechanisms • Supplementary information

Table S1 | Genetic characterization of SCAR16 patients, controls and generated homozygous knockout.
Controls: CO1, CO2, CO3, CO4, CO5, CO6; Patients: STUB1_1, STUB1_2, STUB1_3; f: female; m: male; F: fibroblasts; iPSC: induced pluripotent stem cells; CN: iPSC-derived cortical neurons.

Cell line	Cell type used	Age at skin biopsy, sex	Described mutations in *STUB1*	Annotations
CO1	F	28, m	None	None
CO2	F	24, f	None	None
CO3	F	22, m	None	None
CO4	iPSC, CN	46, f	None	None
CO5	iPSC, CN	37, f	None	None
CO6	iPSC, CN	46, m	None	None
STUB1_1	F, iPSC, CN	17, m	c.367C>G, p.L123V (homozygous)	symptoms: ataxia, spasticity
STUB1_2	F, iPSC, CN	32, m	c.355C>T, p.R119*; c.880A>T, p.I294T	symptoms: ataxia, spasticity, dementia, epilepsy, hypogonadism
STUB1_3	F, iPSC, CN	20, f	c.433A>C, p.K145Q; c.728C>T, p.P243L	symptoms: ataxia, spasticity, dementia, epilepsy
STUB1(-/-)	iPSC, CN	37, f	c.283-438del, p.V94Afs*5 (homozygous)	CRISPR/Cas9 induced homozygous knockout of *STUB1* isogenic to CO5

Table S2 | qRT-PCR Primer

Target gene	Forward sequence	Reverse sequence
HSP90aa1	GCCCAGAGTGCTGAATACCC	TTAACAGGTGCCCTGCTTCT
HSPA1A/B	GGTGCTGACCAAGATGAAG	CTGCGAGTCGTTGAAGTAG
HSPA8	CCCTTTATGGTGGTGAATGA	GTAACAGTCTTCCCAAGGTAG
HSPB1	AAGCTAGCCACGCAGTCCAA	CGGCAGTCTCATCGGATTTT
HSPB8	GCTTCAAGCCAGAGGAGTTGA	ACAATGCCACCTTCTTGCTGT
HSPA5/BiP	CCCGAGAACACGGTCTTTGA	TTCAACCACCTTGAACGGCA
DNAJB1	CTGTCTTCTCTTTGGCCATCTC	CTGCTGGAACGAGAGGTATTG
STUB1	TCAAGGAGCAGGGCAATCGT	CAGCGGGTTCCGGGTGAT
GAPDH	TCACCAGGGCTGCTTTTAAC	GACAAGCTTCCCGTTCTCAG
TBP	CTTCGGAGAGTTCTGGGATTG	CACGAAGTGCAATGGTCTTTAG

Table S3 | Proteins expressed in at least 2 control CNs and no patient CNs. Values are given in Log10 label-free quantification (LFQ) intensity.

Protein name	CO4	CO5	CO6	STUB1_1	STUB1_2	STUB1_3
AUP1	7,18	7,22	7,19	0	0	0
EBF1/2/3	6,88	7,41	7,04	0	0	0
SEC61A1	7,15	7,11	7,13	0	0	0
FMNL2	7,02	6,99	7,12	0	0	0
EML1	7,05	7,04	6,95	0	0	0
CKMT1A	6,94	7,17	6,85	0	0	0
SMPD3	6,80	7,10	7,04	0	0	0
NDUFC2;NDUFC2-KCTD14	6,92	6,98	7,04	0	0	0
FN3KRP	6,99	7,00	6,89	0	0	0
GABARAPL2	6,98	6,89	6,93	0	0	0
MUC2;MLP	6,83	6,99	6,96	0	0	0
ACTN4	6,94	6,95	6,89	0	0	0
RPS10;RPS10P5	6,81	6,80	6,99	0	0	0

TFAM	6,85	6,87	6,88	0	0	0
STAT5B;STAT5A	6,85	6,78	6,81	0	0	0
EVI5L	6,78	6,93	6,69	0	0	0
STUB1	6,83	6,82	6,73	0	0	0
STX1A	6,78	6,89	6,64	0	0	0
CMIP	6,83	6,82	6,66	0	0	0
TPPP	6,96	6,65	6,57	0	0	0
PSMD9	6,85	6,72	6,67	0	0	0
CELF3	6,69	6,86	6,63	0	0	0
LGALSL	6,67	6,90	6,60	0	0	0
RELN	6,73	6,57	6,86	0	0	0
TMEM65	6,59	6,94	6,52	0	0	0
PTP4A1/2	6,74	6,69	6,72	0	0	0
HSPE1-MOB4;MOB4	6,76	6,77	6,60	0	0	0
LPPR1	6,96	6,49	6,54	0	0	0
MTOR	6,72	6,59	6,80	0	0	0
IMPAD1	6,68	6,70	6,73	0	0	0
BACH2	6,27	7,03	6,36	0	0	0
LSM4	6,74	6,69	6,58	0	0	0
REM2	6,60	6,71	6,69	0	0	0
VPS11	6,65	6,72	6,63	0	0	0
KIAA1279	6,72	6,50	6,74	0	0	0
ASAP1	6,60	6,62	6,73	0	0	0
LUC7L	6,65	6,65	6,66	0	0	0
ARHGAP32	6,67	6,71	6,57	0	0	0
RAP2A/B/C	6,61	6,61	6,61	0	0	0
POU3F1/2/3	6,69	6,50	6,59	0	0	0
PAK7	6,50	6,76	6,46	0	0	0
KALRN	6,53	6,62	6,58	0	0	0
PIK3C2B	6,59	6,63	6,51	0	0	0
INTS1	6,49	6,51	6,69	0	0	0
MAVS	6,68	6,39	6,60	0	0	0
GMPPB	6,64	6,64	6,37	0	0	0
RABL6	6,48	6,61	6,56	0	0	0
EIF3K	6,56	6,55	6,55	0	0	0
CA2	6,48	6,42	6,70	0	0	0
CELSR3	6,49	6,56	6,54	0	0	0
EXOC3	6,50	6,57	6,51	0	0	0
WDR7	6,52	6,59	6,43	0	0	0
CEP97	6,55	6,51	6,48	0	0	0
NDUFA12	6,56	6,55	6,40	0	0	0
CBX1	6,44	6,50	6,55	0	0	0
RPRD2	6,52	6,43	6,49	0	0	0
NACAD	6,51	6,45	6,48	0	0	0
TRAF6	6,51	6,40	6,50	0	0	0
ACSS2	6,61	6,39	6,39	0	0	0
IK	6,37	6,54	6,49	0	0	0
STAU2	6,50	6,53	6,37	0	0	0
ARVCF	6,48	6,54	6,38	0	0	0
STAMBP	6,44	6,49	6,46	0	0	0
AIDA	6,46	6,44	6,43	0	0	0
HSPA14	6,38	6,44	6,50	0	0	0
TIGD4;APBB1	6,44	6,42	6,44	0	0	0
CNOT10	6,40	6,46	6,44	0	0	0
NARS2	6,46	6,43	6,41	0	0	0
INPP5F	6,36	6,36	6,50	0	0	0

SLC35A4	6,38	6,43	6,43	0	0	0
CHERP	6,40	6,41	6,38	0	0	0
C10orf76	6,40	6,45	6,32	0	0	0
AMER2	6,50	6,30	6,31	0	0	0
FAM188A	6,54	6,24	6,29	0	0	0
ARFIP2	6,29	6,43	6,40	0	0	0
PPP1R12A	6,35	6,37	6,40	0	0	0
FMR1	6,46	6,20	6,40	0	0	0
MRE11A	6,35	6,26	6,45	0	0	0
TRAPPC1	6,39	6,40	6,27	0	0	0
POGLUT1	6,33	6,30	6,41	0	0	0
DCK	6,26	6,54	6,15	0	0	0
GABBR1	6,32	6,44	6,26	0	0	0
TTYH1	6,27	6,19	6,51	0	0	0
PML	6,28	6,17	6,51	0	0	0
EXOC6B	6,45	6,28	6,26	0	0	0
CNTNAP5	6,29	6,32	6,36	0	0	0
DIS3L2	6,42	6,23	6,18	0	0	0
CRBN	6,33	6,24	6,19	0	0	0
FYTTD1	6,26	6,35	6,10	0	0	0
DNAJB1	6,30	6,24	6,18	0	0	0
NPC1	6,19	6,15	6,32	0	0	0
TRAPPC9	6,12	6,28	6,13	0	0	0
METTL13	6,27	6,14	6,11	0	0	0
GEMIN5	6,17	6,11	6,21	0	0	0
ACTBL2	9,07	0	7,39	0	0	0
SRSF7	0	7,10	7,10	0	0	0
PCNT	7,13	7,03	0	0	0	0
TMEM35	7,08	0	6,96	0	0	0
ATR	0	6,99	6,98	0	0	0
KIAA1211	7,03	0	6,88	0	0	0
RPL29	6,88	0	7,02	0	0	0
NUDT1	0	7,05	6,80	0	0	0
FAF1	6,93	6,86	0	0	0	0
THG1L	7,04	0	6,68	0	0	0
RPS15A	6,84	6,88	0	0	0	0
NKIRAS2	0	6,91	6,75	0	0	0
PFDN2	6,88	0	6,79	0	0	0
COX5B	6,68	6,89	0	0	0	0
ERCC4	6,73	6,81	0	0	0	0
DNAJC7	6,68	0	6,77	0	0	0
RAP1GAP	6,42	6,88	0	0	0	0
COX5A	6,69	0	6,72	0	0	0
SREK1	0	6,64	6,72	0	0	0
CLVS1	6,83	6,44	0	0	0	0
DAZAP1	0	6,65	6,69	0	0	0
RASAL2	6,58	6,73	0	0	0	0
DHX38	6,56	6,73	0	0	0	0
KCTD15	6,72	0	6,56	0	0	0
SORBS2	6,72	0	6,54	0	0	0
TAB1	6,60	0	6,66	0	0	0
SMAP2	6,66	6,59	0	0	0	0
NR1I2	6,75	0	6,43	0	0	0
GNB4	0	6,79	6,31	0	0	0
SCAPER	6,64	6,59	0	0	0	0
FOXP2	6,76	6,37	0	0	0	0
EPHB1	0	6,68	6,52	0	0	0

PREPL	6,46	6,67	0	0	0	0
CLPB	0	6,60	6,56	0	0	0
SIRPA	0	6,62	6,48	0	0	0
UQCRQ	6,51	0	6,60	0	0	0
RABL2A/B	6,51	6,60	0	0	0	0
RABGEF1	6,54	6,54	0	0	0	0
QTRTD1	6,41	0	6,63	0	0	0
KIAA0930	6,55	0	6,50	0	0	0
AKT3	6,51	0	6,53	0	0	0
SKIV2L	6,42	0	6,60	0	0	0
PPIL4	0	6,52	6,49	0	0	0
RAD23A	6,53	0	6,45	0	0	0
EFNB2	6,53	6,44	0	0	0	0
ZYG11B	6,51	6,46	0	0	0	0
ERI3	6,48	6,49	0	0	0	0
CEND1	6,47	0	6,47	0	0	0
NME7	6,53	6,39	0	0	0	0
SELENBP1	6,58	0	6,29	0	0	0
DIAPH1	6,46	6,45	0	0	0	0
TRMT10C	0	6,44	6,45	0	0	0
TMEM163	6,42	0	6,46	0	0	0
KATNAL1	0	6,32	6,53	0	0	0
DDX39A	0	6,32	6,52	0	0	0
DTX3	6,46	6,40	0	0	0	0
VCPIP1	6,38	6,46	0	0	0	0
DCHS1	6,44	0	6,39	0	0	0
ARMC1	0	6,44	6,36	0	0	0
NDN	0	6,45	6,34	0	0	0
UBR7	0	6,32	6,47	0	0	0
BCS1L	0	6,50	6,26	0	0	0
FECH	6,47	0	6,26	0	0	0
GCLC	6,24	0	6,43	0	0	0
QTRT1	0	6,43	6,22	0	0	0
NAA30	6,25	6,40	0	0	0	0
SMG8	6,29	6,36	0	0	0	0
CHCHD6	0	6,44	6,15	0	0	0
EIF2B2	6,34	0	6,30	0	0	0
PCID2	0	6,23	6,39	0	0	0
MYCBP2	6,28	6,34	0	0	0	0
SLC25A29	6,27	6,33	0	0	0	0
NCOA5	6,20	6,34	0	0	0	0
CBWD1/2/3/5/6/7	6,17	6,36	0	0	0	0
RDH14	0	6,45	5,97	0	0	0
RPS6KA4/5	6,24	6,29	0	0	0	0
PRPF4	6,28	6,24	0	0	0	0
APPL2	6,19	0	6,31	0	0	0
ERCC2	6,29	0	6,21	0	0	0
ELP3	6,21	6,28	0	0	0	0
NAGLU	6,25	6,20	0	0	0	0
ZFYVE20	6,28	0	6,15	0	0	0
KIAA1033	6,01	6,35	0	0	0	0
GAK	0	6,33	6,03	0	0	0
CDH10	6,29	0	6,08	0	0	0
C18orf8	6,21	0	6,19	0	0	0
DOCK3	6,06	6,28	0	0	0	0
CCNY;CCNYL1/2	6,11	0	6,17	0	0	0
IP6K1	6,08	6,14	0	0	0	0
CAND2	0	6,06	6,11	0	0	0
LZTS1	5,97	6,17	0	0	0	0

Table S4 | Proteins expressed in at least 2 patient CNs and no control CNs. Values are given in Log10 label-free quantification (LFQ) intensity.

Protein name	CO4	CO5	CO6	STU B1_ 1	STU B1_ 2	STU B1_ 3
VPS18	0	0	0	6,04	6,06	6,13
SUPV3L1	0	0	0	6,23	6,09	6,07
CPT1C	0	0	0	6,24	6,14	6,17
PTPRO	0	0	0	6,21	6,20	6,18
TARBP2	0	0	0	6,19	6,16	6,25
ADAM10	0	0	0	6,37	6,12	6,06
PRPS2	0	0	0	6,25	6,13	6,23
PIGU	0	0	0	6,43	6,06	6,02
WDR26	0	0	0	6,27	6,18	6,29
DHX16	0	0	0	6,26	6,20	6,32
NUDCD2	0	0	0	6,18	6,35	6,27
STAM2	0	0	0	6,25	6,32	6,28
USP48	0	0	0	6,25	6,29	6,34
FBXO21	0	0	0	6,21	6,33	6,38
MRPS9	0	0	0	6,46	6,20	6,24
CNTN2	0	0	0	6,39	6,41	6,11
TRAF2	0	0	0	6,32	6,37	6,31
THNSL1	0	0	0	6,35	6,32	6,34
FAHD2A/B	0	0	0	6,42	6,29	6,29
SMYD3	0	0	0	6,31	6,17	6,50
Mar-05	0	0	0	6,45	6,26	6,31
CHTOP	0	0	0	6,28	6,39	6,40
UROD	0	0	0	6,45	6,28	6,33
FAR1	0	0	0	6,51	6,23	6,31
DTNA;DTNB	0	0	0	6,52	6,27	6,28
KDM3B	0	0	0	6,42	6,46	6,20
TRAPPC4	0	0	0	6,37	6,42	6,36
ZC3HC1	0	0	0	6,43	6,40	6,32
TOM1L2	0	0	0	6,37	6,34	6,44
MOCS3	0	0	0	6,31	6,45	6,40
MID1	0	0	0	6,66	6,21	6,15
PITPNM1	0	0	0	6,46	6,31	6,45
RELA	0	0	0	6,41	6,43	6,41
SNRPG;SNRPGP1 5	0	0	0	6,42	6,39	6,44
CNOT2	0	0	0	6,37	6,44	6,44
SLC8A2	0	0	0	6,15	6,58	6,44
ALG9	0	0	0	6,55	6,37	6,33
SRPK2	0	0	0	6,36	6,44	6,49
ACSL1	0	0	0	6,54	6,38	6,36
FAM120B	0	0	0	6,44	6,46	6,41
PTCD3	0	0	0	6,58	6,38	6,30
WASF3	0	0	0	6,23	6,59	6,48
RSRC2	0	0	0	6,41	6,60	6,36
PUM2	0	0	0	6,36	6,59	6,44
GMPPA	0	0	0	6,56	6,42	6,45
HMOX2	0	0	0	6,55	6,42	6,50
YARS2	0	0	0	6,64	6,38	6,41
SUMF2	0	0	0	6,63	6,41	6,41

Disease Models & Mechanisms • Supplementary information

DNAJC9	0	0	0	6,60	6,45	6,44
UBE4A	0	0	0	6,46	6,49	6,55
NPC2	0	0	0	6,72	6,34	6,34
TUBGCP2	0	0	0	6,48	6,50	6,54
CYTH1/2/3	0	0	0	6,47	6,57	6,49
CRAT	0	0	0	6,47	6,51	6,55
C8orf82	0	0	0	6,65	6,45	6,40
PFDN4	0	0	0	6,45	6,56	6,54
NOSIP	0	0	0	6,60	6,48	6,47
CNOT11	0	0	0	6,56	6,56	6,46
TMEM11	0	0	0	6,59	6,54	6,44
PACSIN2	0	0	0	6,54	6,48	6,57
ANKRD28	0	0	0	6,30	6,71	6,49
SPTLC1	0	0	0	6,77	6,30	6,39
CYFIP1	0	0	0	6,53	6,47	6,59
RALB	0	0	0	6,51	6,57	6,56
CHID1	0	0	0	6,69	6,45	6,46
ESYT2	0	0	0	6,72	6,41	6,46
C11orf73	0	0	0	6,40	6,46	6,75
CARS2	0	0	0	6,67	6,40	6,60
IGF1R	0	0	0	6,75	6,48	6,40
SELT	0	0	0	6,59	6,57	6,59
BLVRB	0	0	0	6,58	6,67	6,51
SLC35F6	0	0	0	6,82	6,43	6,40
NUP35	0	0	0	6,68	6,54	6,57
TMOD3	0	0	0	6,81	6,42	6,46
ABI1	0	0	0	6,52	6,68	6,59
LIG3	0	0	0	6,65	6,62	6,55
MAP7D1	0	0	0	6,51	6,67	6,63
TMEM256	0	0	0	6,78	6,49	6,48
HRSP12	0	0	0	6,73	6,54	6,54
NME4	0	0	0	6,93	6,36	6,16
DST	0	0	0	6,69	6,60	6,54
DVL2/3	0	0	0	6,69	6,56	6,60
CDK9	0	0	0	6,66	6,58	6,63
FAM171A2	0	0	0	6,54	6,69	6,65
DDI2	0	0	0	6,81	6,49	6,52
UBXN6	0	0	0	6,58	6,67	6,65
AP3B1	0	0	0	6,91	6,42	6,35
COLGALT1	0	0	0	6,92	6,36	6,40
SNX3	0	0	0	6,69	6,59	6,68
HPCAL4	0	0	0	6,47	6,78	6,69
TRAPPC3	0	0	0	6,57	6,67	6,74
C4orf27	0	0	0	6,63	6,67	6,72
AVIL	0	0	0	6,59	6,76	6,66
MOB1A/B	0	0	0	6,94	6,51	6,42
STK4	0	0	0	6,69	6,64	6,73
RBBP7	0	0	0	6,58	6,53	6,88
DENR	0	0	0	6,66	6,77	6,65
WRNIP1	0	0	0	6,65	6,63	6,80
TMCO1	0	0	0	6,74	6,71	6,67
PPP1R8	0	0	0	6,69	6,70	6,75
GOLGA4	0	0	0	6,60	6,62	6,89
LRPAP1	0	0	0	6,99	6,53	6,44
M6PR	0	0	0	6,80	6,66	6,74
SERINC1	0	0	0	6,86	6,68	6,65
TIGAR	0	0	0	6,77	6,70	6,76

Disease Models & Mechanisms • Supplementary information

RER1	0	0	0	6,93	6,62	6,61
PCYOX1L	0	0	0	6,71	6,77	6,78
GPC6	0	0	0	6,98	6,56	6,59
TIMM23;TIMM23B	0	0	0	6,73	6,79	6,78
PSEN1/2	0	0	0	6,95	6,68	6,66
MBOAT7	0	0	0	6,94	6,70	6,67
PPAPDC2	0	0	0	6,69	6,93	6,72
SYNE1	0	0	0	6,88	6,72	6,76
PEX11B	0	0	0	6,88	6,77	6,79
PHPT1	0	0	0	6,79	6,81	6,87
CARHSP1	0	0	0	6,84	6,86	6,85
NDUFS7	0	0	0	6,97	6,80	6,81
BDH2	0	0	0	6,92	6,85	6,83
PDPK1/2	0	0	0	6,76	6,99	6,83
REEP5	0	0	0	6,96	6,84	6,81
ITGB1	0	0	0	7,22	6,62	6,56
UNC119B	0	0	0	6,93	6,84	6,97
MMAB	0	0	0	7,11	6,94	6,73
NELFA	0	0	0	6,77	7,05	7,01
POFUT1	0	0	0	7,23	6,71	6,74
CISD2	0	0	0	6,95	6,91	7,03
RBM8A	0	0	0	6,97	7,01	6,96
C21orf33	0	0	0	7,06	7,05	7,06
SSR4	0	0	0	7,23	7,02	6,91
SEC61B	0	0	0	7,17	7,09	6,99
ARSB	0	0	0	7,32	6,94	7,03
PAPSS1	0	0	0	7,33	7,11	7,05
NR2F1	0	0	0	7,70	7,20	6,85
RTN1	0	0	0	7,31	7,67	7,53
LMNA	0	0	0	8,02	6,58	6,39
ACTB	0	0	0	8,12	8,18	8,14
MRPS34	0	0	0	0	6,08	6,01
KATNB1	0	0	0	5,99	0	6,12
PI4K2A	0	0	0	6,23	5,79	0
C7orf26	0	0	0	6,10	6,11	0
PICK1	0	0	0	0	6,10	6,17
NDC1	0	0	0	6,24	0	6,21
EFHD1/2	0	0	0	0	6,33	6,11
FOXRED1	0	0	0	6,26	6,21	0
IQSEC1	0	0	0	6,11	6,36	0
UBE4B	0	0	0	0	6,16	6,34
MPP6	0	0	0	6,17	6,34	0
PTPRD	0	0	0	0	6,29	6,23
TMEM68	0	0	0	6,35	6,18	0
RBM17	0	0	0	6,29	0	6,27
EI24	0	0	0	6,28	6,31	0
PLCD1	0	0	0	6,37	0	6,21
TPD52L2	0	0	0	6,43	0	6,18
GTF3C5	0	0	0	6,36	6,28	0
ARMC10	0	0	0	6,35	6,31	0
AASS	0	0	0	6,46	6,18	0
TAOK1	0	0	0	0	6,39	6,36
EXOSC7	0	0	0	6,35	0	6,40
RFX3	0	0	0	6,28	6,45	0
PEX14	0	0	0	6,34	0	6,42
MRPL48	0	0	0	6,52	6,21	0
ABCF2	0	0	0	6,49	6,29	0

MYT1	0	0	0	0	6,32	6,47
TRMT1;SEMA4B	0	0	0	6,49	0	6,30
AGL	0	0	0	0	6,50	6,30
H6PD	0	0	0	6,50	0	6,31
RNF214	0	0	0	0	6,43	6,40
GMPR2	0	0	0	6,27	0	6,53
DAGLB	0	0	0	6,61	0	6,08
ARRB2	0	0	0	0	6,46	6,39
MRPL4	0	0	0	6,49	6,36	0
EIF4G3	0	0	0	6,44	0	6,42
ARHGAP21	0	0	0	0	6,42	6,45
MTPAP	0	0	0	6,60	0	6,19
NUBP2	0	0	0	6,33	0	6,53
CPOX	0	0	0	6,46	0	6,43
RAB3B	0	0	0	6,55	6,32	0
MPI	0	0	0	6,36	0	6,55
TMEM214	0	0	0	6,67	0	6,06
RMDN1	0	0	0	6,56	0	6,37
HIBADH	0	0	0	6,67	6,12	0
SEH1L	0	0	0	6,57	6,36	0
MRPL43	0	0	0	0	6,44	6,51
NOL4L	0	0	0	0	6,43	6,52
PJA2	0	0	0	6,54	6,42	0
CELF4	0	0	0	0	6,45	6,51
ERLIN1	0	0	0	6,62	0	6,30
DNMT1	0	0	0	6,59	0	6,40
TSPAN6	0	0	0	6,69	0	6,21
SGSH	0	0	0	6,69	6,22	0
RAP1A/B	0	0	0	6,49	0	6,57
FDXR	0	0	0	6,70	6,29	0
CHL1	0	0	0	6,71	6,32	0
MACROD2	0	0	0	0	6,55	6,60
GUCY1B3	0	0	0	0	6,52	6,64
SPCS1	0	0	0	0	6,62	6,59
TMEM30A	0	0	0	0	6,70	6,51
PPP4R1	0	0	0	0	6,59	6,68
EIF2B5	0	0	0	6,58	0	6,69
RPS15	0	0	0	0	6,72	6,56
CCDC132	0	0	0	0	6,62	6,67
PRNP	0	0	0	0	6,62	6,71
PLOD3	0	0	0	6,88	6,22	0
RAB33A	0	0	0	0	6,72	6,64
TOMM20	0	0	0	0	6,77	6,58
ITGAV	0	0	0	6,90	0	6,28
SF3B4	0	0	0	6,68	0	6,73
CTSA	0	0	0	6,84	6,59	0
CPVL	0	0	0	6,92	0	6,47
RBX1	0	0	0	0	6,83	6,67
FAT1	0	0	0	6,92	0	6,55
EIF1;EIF1B	0	0	0	6,78	6,77	0
BTF3L4	0	0	0	6,73	6,82	0
PVRL2	0	0	0	6,97	0	6,50
BSCL2	0	0	0	6,90	6,67	0
VPS52	0	0	0	6,83	0	6,91
EPHA7	0	0	0	7,05	6,69	0
GPM6B	0	0	0	6,87	7,12	0
SCGN	0	0	0	0	6,60	7,28

Disease Models & Mechanisms • Supplementary information

MGST1	0	0	0	7,30	0	6,75
DBI	0	0	0	0	7,12	7,14
PDPR	0	0	0	0	7,07	7,19
UBTF	0	0	0	7,19	0	7,22
P4HA1	0	0	0	7,50	6,28	0
TP53I11	0	0	0	0	7,41	7,09
SWAP70	0	0	0	7,46	7,29	0
CALB1	0	0	0	7,80	6,78	0
TTN	0	0	0	7,99	8,04	0
